# Physiological regulation underlying the alleviation of cadmium stress in maize seedlings by exogenous glycerol

**DOI:** 10.1038/s41598-025-94385-4

**Published:** 2025-04-01

**Authors:** Qiao Li, Chunda Niu, Jiaxu Guo, Geng Chen, Jingti Li, Lei Sun, Wei Li, Tianpu Li

**Affiliations:** 1 College of Agriculture, Northeast Agricultural University, Harbin, 150030 China; 2 College of Resources and Environment, Northeast Agricultural University, Harbin, 150030 China; 3 College of Arts and Sciences, Northeast Agricultural University, Harbin, 150030 China

**Keywords:** Maize, Cadmium stress, Glycerol, Physiological characteristics, Transcriptome, Plant sciences, Plant physiology

## Abstract

Cadmium (Cd) contamination in maize poses a significant threat to global food security due to its persistent accumulation in crops. In this study, the effects of foliar application of glycerol on Cd accumulation in maize seedlings were studied. Our results demonstrated that under Cd treatment, biomass, total chlorophyll content, net photosynthetic rate (*Pn*), Ribulose-1,5-bisphosphate carboxylase/oxygenase (*RuBisCO*) activity, Phosphoenolpyruvate carboxylase (*PEPC*) activity, sucrose levels, and carbohydrate levels in maize seedlings significantly increased after glycerol application. H_2_O_2_ and MDA levels in both the aboveground and belowground portions of the maize plants significantly decreased. Moreover, superoxide dismutase (SOD), peroxidase (POD), and catalase (CAT) activities in the aboveground parts significantly increased. Notably, maize plants used glycerol to chelate Cd, which was fixed within the cell wall and soluble fraction of the roots, reducing Cd transport to the shoots and significantly lowering the Cd transport coefficient (TF). Transcriptomic data suggested that glycerol-mediated alleviation of Cd stress in maize seedlings may be associated with phenylpropanoid biosynthesis, plant-pathogen interactions and photosynthesis pathways. These molecular patterns align with the observed physiological improvements. This study provided a novel approach to effectively alleviate excessive Cd in maize and suggested possible applications of glycerol in cultivating plant resistance to heavy metals.

**Supplementary Information:**

The online version contains supplementary material available at 10.1038/s41598-025-94385-4.

## Introduction

Cadmium (Cd) pollution has become a prominent environmental problem worldwide. According to the 2014 National Soil Pollution Survey Bulletin, the inorganic pollutant Cd has the highest pollution level with an exceedance rate of 7.0%^1^. Water bodies become enriched with Cd in different ways, and Cd eventually enters farmlands through irrigation and other means. Cd can persist in soil for a long time, and crops absorb soil nutrients while absorbing Cd, leading to a large amount of Cd enrichment in crops^2,3^. Consequently, Cd enters the human body through the food chain, causing severe harm to the human body. Maize (*Zea mays* L.) is one of the most widely planted crops in the world and one of the most important grain crops, feed crops and economic crops in China^4^. Compared with other grain crops, maize has a greater ability to absorb Cd and transport it to plant shoots, resulting in Cd accumulation and affecting plant growth^5^. It has been reported that Cd treatment significantly inhibites the growth of maize seedling, as the cell membrane system is damaged and organelles are severely affected^6^. Because it is difficult to remove Cd from polluted farmland^7^, finding an effective strategy to mitigate the accumulation of Cd in maize is the key in reducing its impacts on food security.

Previous studies have shown that the application of exogenous substances can increase plants’ tolerance to Cd and reduce the damage caused by Cd in plant cells^8^. Exogenous melatonin has been shown to effectively increase the activity of antioxidant enzymes and the expression of antioxidant-related genes in crops under Cd stress^9^. Moreover, various exogenous additives decrease the Cd content in wheat, resulting in reduced Cd uptake^10^. Glycerol (Gly) is a substance that is used for energy production; it is colorless and odorless and has strong economic value^11^. As a three-carbon molecule, glycerol not only provides a carbon skeleton for cell growth and metabolic activities but also plays an important role in photosynthesis and plant stress resistance^12^. It has been reported that foliar spray of glycerol enhances cocoa plant’s stress resistance against diseases^13^. Similarly, foliar application of glycerol at the filling stage was found to reduce the Cd content in brown rice and increase the growth of rice under Cd pollution in the field^14^. Given that maize possesses unique C4 photosynthetic pathways and vascular bundle structures that may differentially regulate Cd translocation, investigating the Cd mitigation effects of glycerol in maize is of significant importance. However, current research on the application of glycerol in the C4 graminaceous crop maize remains limited, and the underlying mechanisms require further exploration.

In this study, we designed a pot experiment to analyze the changes in morphological traits, physiological characteristics and molecular responses of maize seedlings following the application of exogenous glycerol during the second week (14 days) and third week (21 days) of Cd stress. This study aims to reveal the physiological and molecular patterns of maize seedlings in response to Cd stress under the influence of exogenous glycerol to develop safer, more effective and more economical methods for safe crop production. We hypothesized that (1) the addition of exogenous glycerol as a carbon skeleton can improve the photosynthetic performance and antioxidant capacity of maize seedlings and that (2) exogenous glycerol reduces Cd accumulation by regulating maize photosynthesis and the expression levels of stress-related pathways and resistance genes.

## Methods

### Experimental design

This experiment was carried out in the Laboratory of Maize Cultivation Physiology, College of Agriculture, Northeast Agricultural University, Heilongjiang Province, China (45°72’ to 45°74’ N; 126°71’ to 126°73’ E). The tested seed was Zhengdan 958, which was selected by the Institute of Grain Crops, Henan Academy of Agricultural Sciences. The source of purchase was Kenfeng Seed Industry Co., Ltd. The seeds were disinfected with 1% NaClO for 5 min and rinsed with distilled water repeatedly. The treated seeds were placed in a germination box with moist filter paper and subsequently transferred to an artificial climate chamber for dark germination for 24 h. After the seeds had germinated, seedlings with consistent growth were selected and transplanted into plastic boxes (16 cm long, 5 cm wide, and 20 cm high) containing vermiculite. Each box was allocated to one seedling. The temperature in the greenhouse was maintained at 22–26 °C, the light intensity was set at 400 µmol/(m^2^·s) PAR, and the relative humidity was approximately 70%. The seedlings were grown under a cycle of 16 h of light and 8 h of darkness. Cadmium chloride (CdCl_2_) was dissolved in Hoagland nutrient solution to prepare solutions with Cd concentrations of 400 µM and 800 µM. After 5 days of maize growth, the maize seedlings were irrigated with Cd-containing nutrient solution every other day, with each irrigation consisting of 80 mL, for a total of 10 times. Concurrently, 5 mL of distilled water or 5 mL of 1 mM glycerol was sprayed on the leaves every 2 days for a total of 10 times. The glycerol concentration was determined on the basis of our preliminary experiments. The experiment involved six different treatments: (1) a Cd concentration of 0 µM and foliar application of distilled water (CK); (2) a Cd concentration of 0 µM and foliar application of 1 mM glycerol (Gly); (3) a Cd concentration of 400 µM and foliar application of distilled water (400Cd); and (4) a Cd concentration of 400 µM and foliar application of 1 mM glycerol (400Cd + Gly). (5) Cd concentration 800 µM and foliar application of distilled water (800Cd); 6) Cd concentration 800 µM and foliar application of 1 mM glycerol (800Cd + Gly). Each treatment was repeated 12 times.

The maize seedlings were harvested after 14 and 21 days of Cd stress. After impurities were removed, the fresh leaves and root tissues were quickly frozen in liquid nitrogen and stored at -80 °C for subsequent analyses.

### Determination of biomass and morphological indices

After 14 and 21 days of treatment, the shoots and roots were placed in an oven at 105 °C for 30 min, after which the oven temperature was adjusted to 80 °C until the sample was completely dry. The weights of the two parts were measured separately. The root images were obtained using a Midcrystal i800 Plus root scanner, and the root morphology was analyzed using the Hangzhou Wanshen LA-S root analysis system.

### Determination of physiological indices

The total chlorophyll content in the maize leaves was determined according to the methods of Lichtenthaler^15^. Fresh leaves (0.2 g) were extracted in 90% acetone solution for 24 h. Afterward, the absorbance was measured with a UV-spectrophotometer at wavelengths of 663.2 nm and 646.8 nm, respectively. Finally, the total chlorophyll content was calculated.

The net photosynthetic rate (*Pn*) of the second mature maize leaf was measured by a portable photosynthetic measurement system. The light intensity in the leaf compartment was 1600 µmol·m^-2^·s^-1^ PAR, and the greenhouse carbon dioxide concentration was approximately 450 µM.

Fresh corn leaves (0.2 g) were placed in a precooled mortar, a phosphate buffer solution a pH of 8.2 was added, the homogenate was fully ground and centrifuged at 4 °C and 15,000 r for 10 min, after which the supernatant was collected. An enzyme-linked immunosorbent assay (ELISA, Wuhan Purity Biotechnology Co., Ltd.) was used to determine the activity of the Ribulose-1,5-bisphosphate carboxylase/oxygenase (*RuBisCO*) and Phosphoenolpyruvate carboxylase (*PEPC*).

The sucrose content in the maize leaves was determined as described by Muneer^16^. A 0.1 g dried corn leaf sample that was previously ground and sieved was weighed into a centrifuge tube. Ethanol (90%, 10 mL) was added, and the tube was incubated in a water bath at 60 °C for 1 h. After incubation, 15 mL of 95% ethanol was added to bring the total solution volume to 25 mL. One milliliter of an aliquot was transferred to a new tube, and 1 mL of 5% phenol reagent was added. The solution was mixed thoroughly, and then 5 mL of concentrated sulfuric acid was added. After the solution was allowed to cool to room temperature, the absorbance was measured at a wavelength of 485 nm using an ultraviolet spectrophotometer. The corresponding sucrose concentration was then calculated on the basis of a standard curve, which was prepared with glucose solutions as a reference.

The carbohydrate content was determined via the anthrone method^17^. Dry leaf tissue (0.1 g) was added to a boiling tube. The tissue was hydrolyzed in 5 mL of 2.5 N-HCl by boiling in a water bath for 3 h. After the tube was cooled to room temperature, the solid sodium carbonate in the solution was neutralized by adding sulfuric acid until bubbling ceased. The sample was then centrifuged, and 1 mL of the supernatant was mixed with 4 mL of anthrone reagent. The mixture was heated in a boiling water bath for 8 min and then cooled to room temperature. The absorbance of the solution was measured at 630 nm using a UV‒Vis spectrophotometer. The carbohydrate concentration was determined on the basis of a standard curve, which was prepared with glucose solutions as the standard.

Fresh tissue (0.1 g) was ground into a homogenate in 1 mL of cold TCA (0.1%). The content of hydrogen peroxide (H_2_O_2_) was determined by the method of Murphy and Noack^18^. The malondialdehyde (MDA) content in the maize leaves was measured via the thiobarbituric acid (TBA) method^19^ to evaluate membrane lipid peroxidation.

Fresh maize leaves and roots (0.25 g) were placed into a mortar, and the samples were treated with liquid nitrogen, followed by the addition of 10 mL of cold phosphoric acid buffer (pH 7.8) as a homogeneous mixture for full grinding. The homogenate was centrifuged at 4 °C and 10,000 × g for 20 min, and the superoxide dismutase (SOD), peroxidase (POD), and catalase (CAT) activities were determined according to the methods of Rizwan^20^.

### Determination of the cd content, cd distribution in subcellular fractions and cd transport coefficient

Leaves and roots were cleaned with CaCl_2_ solution and deionized water to remove impurities and metal ions. A total of 0.5 g of the crushed dry sample was transferred to a 50 mL centrifuge tube, and the sample was digested and extracted at a 5:1 ratio of HNO_3_/H_2_O_2_ in a microwave digestion extraction system (ETHOS UP, Milestone, Italy). The concentration of Cd in the solution was subsequently determined by an inductively coupled plasma mass spectrometer (7800 ICP-MS, Agilent, America). Fresh corn leaves and roots (0.2 g) were placed in a precooled mortar, 5 mL of buffer was added, and the mixture was fully ground. The samples were separated into cell wall, organelle and soluble fractions through differential centrifugation. Nitric acid was added to each component for microwave digestion, and the content of Cd in each component was determined by inductively coupled plasma‒mass spectrometry. The Cd translocation factor (TF) was obtained by dividing the Cd content in the aboveground part of the maize seedlings by the Cd content in the underground part.

### Transcriptome analysis

#### RNA extraction from maize leaves

After 21 days of Cd stress, each treatment was biologically repeated three times. Approximately 0.3 g of maize seedling leaves was removed from a 2 mL centrifuge tube and rapidly frozen in liquid nitrogen. The samples were packaged on dry ice and subsequently sent to Shanghai Parsons Biotechnology Co., Ltd., for RNA extraction and determination. RNA integrity was detected by agarose gel electrophoresis and an Agilent 2100 Bioanalyzer.

#### Construction and quality control of the maize leaf cDNA library

Oligo (dT) magnetic beads were used to enrich mRNAs with polyA structures in total RNA, and the RNA was fragmented to 200–300 bp by ion interruption. Using RNA as a template, first-strand cDNA was synthesized with random 6-base primers and reverse transcriptase, and second-strand cDNA was subsequently synthesized with first-strand cDNA as a template. When the second-strand cDNA was synthesized, the base T was replaced by the base U to construct a chain-specific library. After library construction was completed, the library fragments were enriched via PCR amplification, and the library size ranged from 300 to 400 bp. The size of the library was detected by an Agilent 2100 Bioanalyzer, and the total concentration of the library was measured through fluorescence quantitative detection.

#### Transcriptome analysis of maize leaves

The Illumina HiSeq platform was used for sequencing, and paired-end (PE) sequencing was performed on the library. The raw reads obtained from sequencing were processed to eliminate sequencing adapters and primer sequences and to filter out low-quality sequences, thereby obtaining high-quality clean reads that ensure the integrity of the data. The reference genes, gene expression difference analysis, GO enrichment analysis and KEGG enrichment analysis results were compared with those of the Chen Zhe method^21^.

### Data analysis

Statistical analysis were performed using SPSS 25.0 software. All data were presented as mean ± standard error of mean (SEM). One-way analysis of variance was used to compare morphological, physiological and transcriptomic data between treatment groups. When a main effect was observed, a Duncan’s new multiple range test was performed to determine the differences. Values of *p* < 0.05 were regarded as statistically significant. All the data in this study were graphed with Origin 9.0 software.

## Results

### Responses of maize seedling biomass and morphological indices

**Fig. 1:**
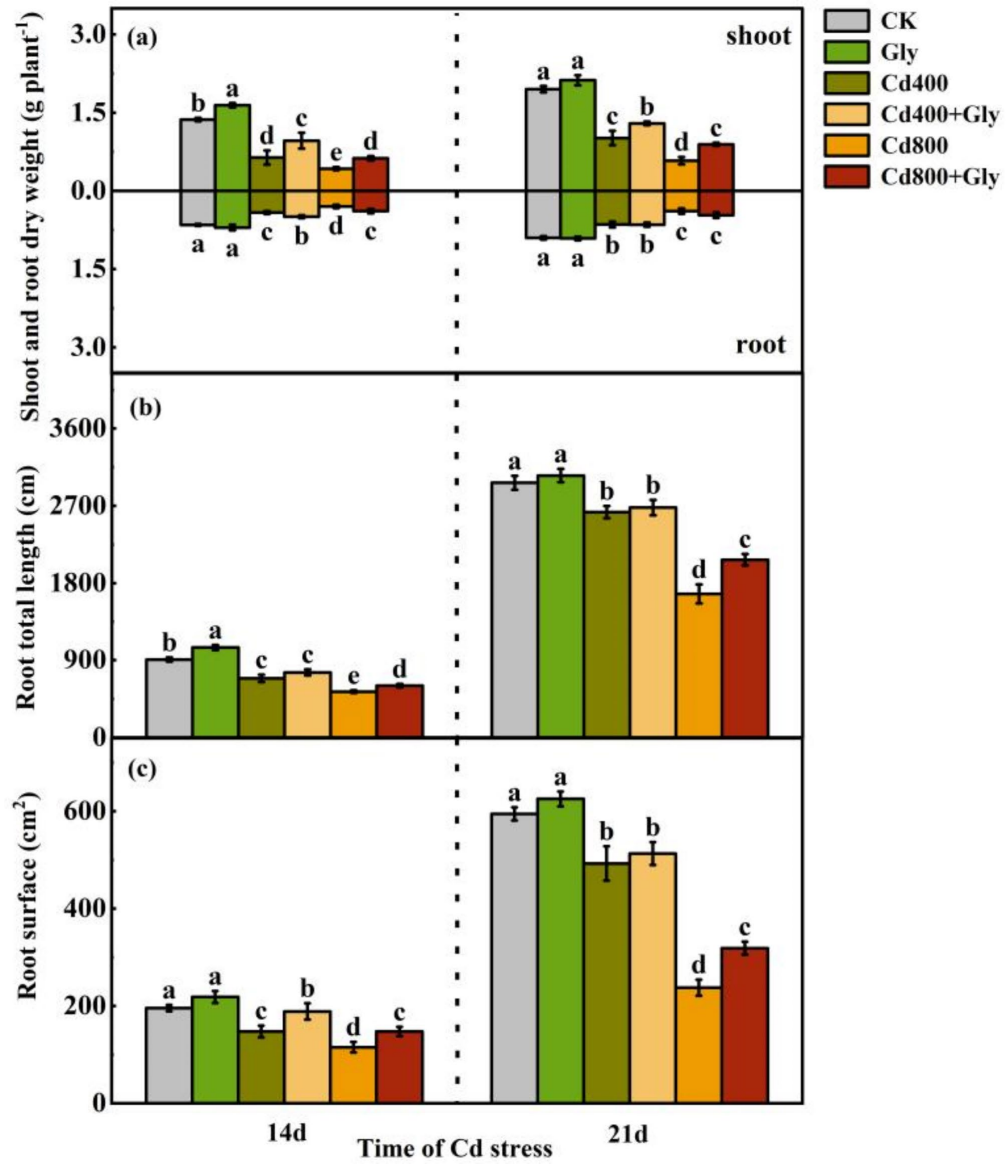
Effects of exogenous glycerol on the morphological indices of maize seedlings under Cd stress. Different lowercase letters indicate that there are significant differences between different treatments at the same growth period at the level of *p* = 0.05, the same letter means no significant difference (*p* > 0.05), while different letters mean significant difference(*p* < 0.05). (**a**) Shoot and root dry weight of maize seedlings under different treatment conditions (g plant^− 1^), (**b**) Root total length of maize seedlings under different treatment conditions (cm), (**c**) Root surface of maize seedlings under different treatment conditions (cm^2^). Data were presented as mean ± standard error of mean (SEM). One-way ANOVA. Post hoc Duncan’s analysis. Values of *p* < 0.05 were regarded as statistically significant.

Compared with the CK treatment, the foliar application of glycerol under normal conditions increased the dry weight of the maize shoots and roots. After the 400 µM and 800 µM Cd treatments, the shoot dry weight and root dry weight of the maize seedlings were significantly lower than those of the CK and Gly plants after 14 and 21 days, respectively, of treatment, and the higher the Cd concentration was, the greater the degree of maize growth inhibition. At 14 d and 21 d, compared with that in the Cd400 treatment, the shoot dry weight in the Cd400 + Gly treatment significantly increased by 50.08% and 27.57%, respectively. Compared with that in the Cd800 treatment, the shoot dry weight in the Cd800 + Gly treatment significantly increased by 46.39% and 54.13%, respectively (Fig. 1a).

Compared with those in the CK treatment, the total length and surface area of the roots in the two Cd concentration groups were significantly lower. However, the application of exogenous glycerol could alleviate this inhibitory effect. At 14 d, the root surface area in the Cd400 + Gly treatment group was significantly greater (by 28.05%) than that in the Cd400 treatment group. Similarly, compared with those in the Cd800 treatment, the total length and surface area of the roots in the Cd800 + Gly treatment significantly increased by 12.94% and 28.01%, respectively. At 21 d, compared with those in the Cd800 treatment, the total length and surface area of the roots in the Cd800 + Gly treatment significantly increased by 12.94% and 28.01%, respectively (Fig. 1b and c).

### Response of physiological indices of maize seedlings

**Fig. 2:**
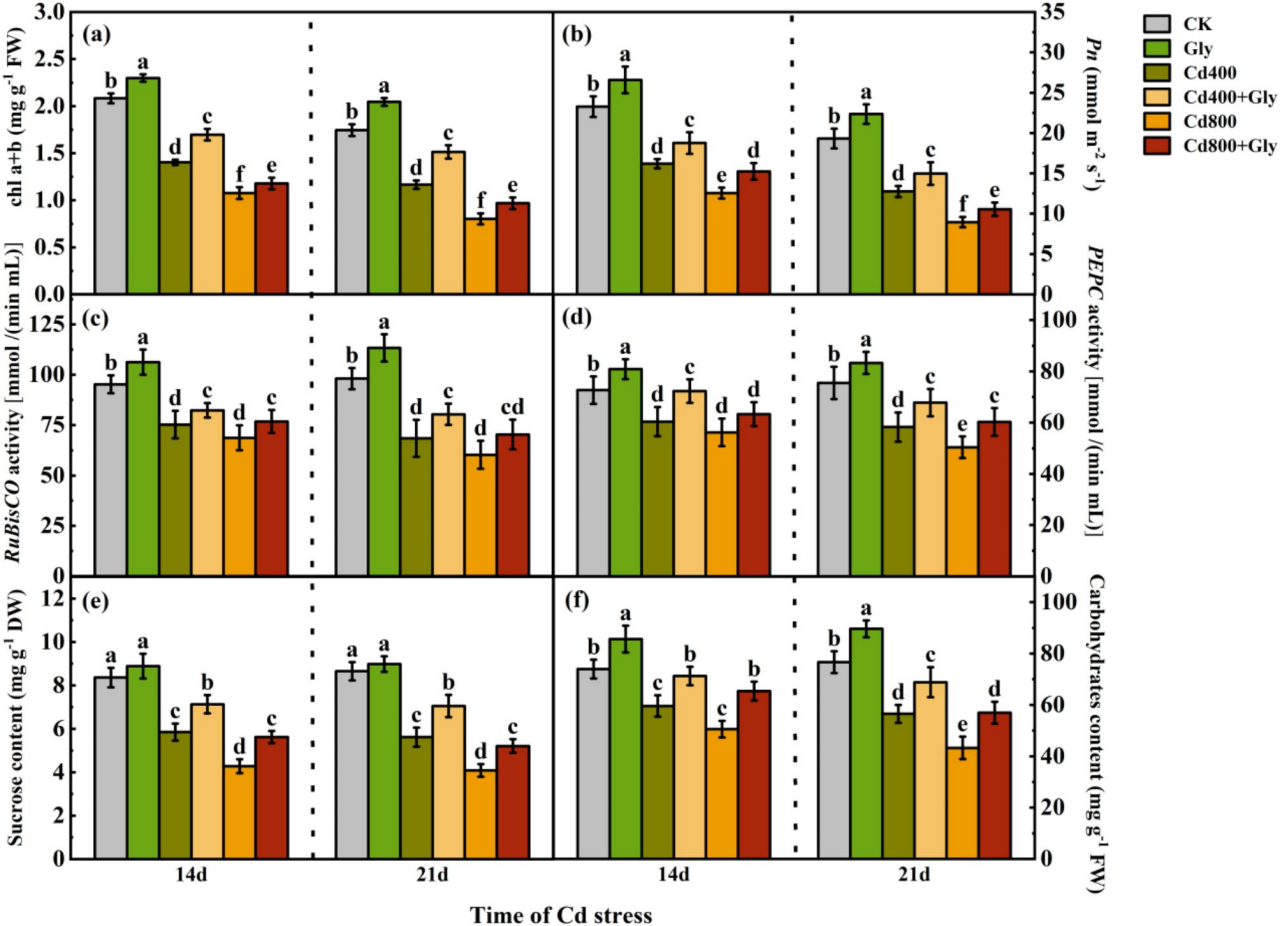
Effects of exogenous glycerol on the photosynthetic performance and photosynthetic products of maize seedlings under Cd stress. Different lowercase letters indicate that there are significant differences between different treatments at the same growth period at the level of *p* = 0.05, the same letter means no significant difference (*p* > 0.05), while different letters mean significant difference(*p* < 0.05). (**a**) chl a + b of maize seedlings under different treatment conditions (mg g^− 1^ FW), (**b**) *Pn* of maize seedlings under different treatment conditions (mmol m^− 2^s^− 1^), (**c**) *RuBisCO* activity of maize seedlings under different treatment conditions [mmol/(min mL)], (**d**) *PEPC* activity of maize seedlings under different treatment conditions [mmol/(min mL)], (**e**) Sucrose content of maize seedlings under different treatment conditions (mg g^− 1^ DW), (**f**) Carbohydratcs content of maize seedlings under different treatment conditions (mg g^− 1^ FW). Data were presented as mean ± standard error of mean (SEM). One-way ANOVA. Post hoc Duncan’s analysis. Values of *p* < 0.05 were regarded as statistically significant.

At 14 d and 21 d, the photosynthesis-related indices of the maize plants exhibited the same trend under each treatment. Compared with those in the CK treatment, the photosynthetic indices in the exogenous glycerol treatment significantly increased under normal conditions, whereas the photosynthesis-related indices decreased with increasing Cd concentration under Cd stress. At 14 d, compared with those in the Cd400 treatment, the total chlorophyll content, *Pn*, *RuBisCO* activity and *PEPC* activity in the Cd400 + Gly treatment significantly increased by 20.87%, 15.84%, 9.43% and 19.67%, respectively. Compared with those in the Cd800 treatment, the total chlorophyll content, *Pn*, and *RuBisCO* activity in the Cd800 + Gly treatment significantly increased by 9.48%, 21.40%, and 11.75%, respectively. However, exogenous glycerol increased *PEPC* activity, but the difference was not significant. At 21 d, compared with those in the Cd400 + Gly treatment, the above indices in the Cd400 + Gly treatment significantly increased by 29.82%, 17.42%, 17.29% and 16.33%, respectively. Compared with those in the Cd800 treatment, the above indices in the Cd800 + Gly treatment significantly increased by 20.57%, 17.91%, and 19.63%, respectively. In addition, the *RuBisCO* activity increased by 16.77%, but the difference was not significant (Fig. 2a, b, c and d).

At 14 d and 21 d, compared with the CK treatment, the Gly treatment increased the sucrose content of the maize shoots, but the difference was not significant. Compared with that in the CK treatment, the shoot sucrose content in the Cd400 and Cd800 treatments was significantly lower (by 30.02% and 48.80%, respectively) at 14 d and significantly lower (by 35.02% and 53.00%, respectively) at 21 d. Compared with that in the same concentration of Cd (400 µM and 800 µM), the sucrose content in the shoots in the Cd400 + Gly treatment and Cd800 + Gly treatment significantly increased, and the sucrose content in the shoots at 14 d increased by 21.88% and 31.31%, whereas that at 21 d increased by 25.44% and 27.38%, respectively (Fig. 2e). Compared with those in the CK treatment, the total carbohydrate content in the maize shoots in the Gly treatment group was significantly greater at 14 d and 21 d, with increases of 15.82% and 16.95%, respectively. Compared with those in the Cd400 treatment, the total carbohydrate content in the Cd400 + Gly treatment significantly increased by 19.59% and 17.84% at 14 d and 21 d, respectively. Compared with those in the Cd800 treatment, the total carbohydrate content in the maize shoots in the Cd800 + Gly treatment also significantly increased, by 29.30% and 31.79% at 14 d and 21 d, respectively (Fig. 2f).

**Fig. 3:**
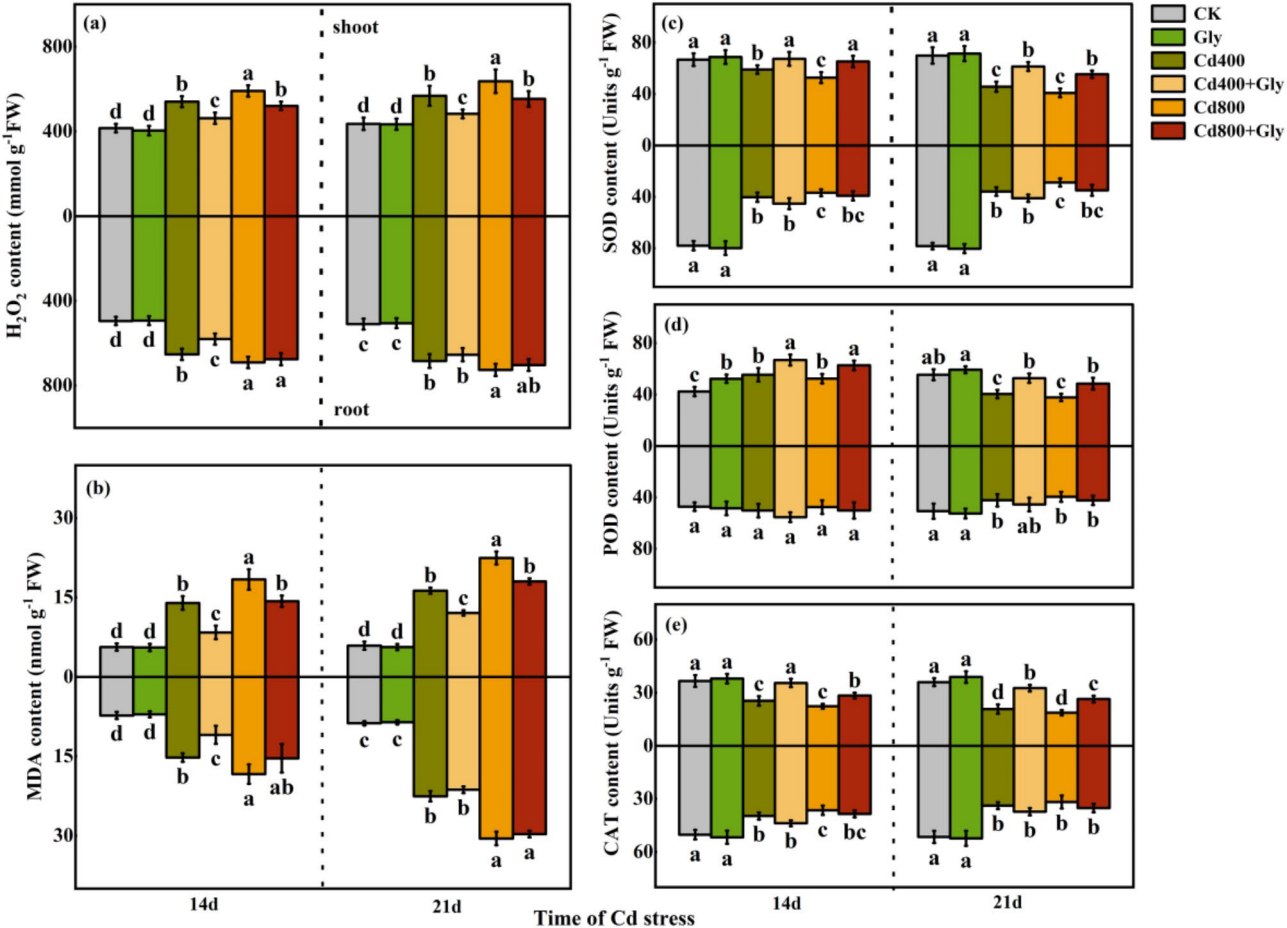
Effects of exogenous glycerol on the antioxidant enzyme activities of maize seedlings under Cd stress. Different lowercase letters indicate that there are significant differences between different treatments at the same growth period at the level of *p* = 0.05, the same letter means no significant difference (*p* > 0.05), while different letters mean significant difference(*p* < 0.05). (**a**) H_2_0_2_ content of maize seedlings under different treatment conditions (mmol g^-1^ FW), (**b**) MDA content of maize seedlings under different treatment conditions (nmol g^-1^ FW), (**c**) SOD content of maize seedlings under different treatment conditions (Units g^-1^ FW), (**d**) POD content of maize seedlings under different treatment conditions (Units g^-1^ FW), (**e**) CAT content of maize seedlings under different treatment conditions (Units g^-1^ FW). Data were presented as mean ± standard error of mean (SEM). One-way ANOVA. Post hoc Duncan’s analysis. Values of *p* < 0.05 were regarded as statistically significant.

Cd stress can lead to the accumulation of large amounts of reactive oxygen species, including hydrogen peroxide (H_2_O_2_) and superoxide anion (O_2_^·-^), in cells, possibly leading to cell damage or death. Under normal conditions, there was no significant difference in the effect of foliar application of glycerol on the H_2_O_2_ content in shoots and roots compared with that in the CK treatment. Compared with the CK treatment, the Cd400 treatment and Cd800 treatment significantly increased the H_2_O_2_ content in the shoots and roots, and the H_2_O_2_ content in the roots was slightly greater than that in the shoots. At 14 d and 21 d of Cd stress, the H_2_O_2_ content in the shoots of the plants in the Cd400 + Gly treatment was significantly lower than that in the shoots in the Cd400 treatment by 14.52% and 15.04%, respectively, and that in the Cd800 + Gly treatment was significantly lower than that in the Cd800 treatment by 11.89% and 13.02%, respectively. The change in H_2_O_2_ content in the roots of the plants in each treatment group was consistent with the change in H_2_O_2_ content in the shoots. Only the H_2_O_2_ content in the Cd400 + Gly treatment group was significantly lower than that in the Cd400 treatment group at 14 d (Fig. 3a).

Malondialdehyde is a product of membrane lipid peroxidation, and its content can indicate damage to the cell membrane. Cd stress led to a large accumulation of MDA in the shoots and roots. With prolonged Cd stress, the content of MDA in shoots and roots tended to increase, and the content of MDA in roots was greater than that in shoots. At 14 d and 21 d, the MDA content in the shoots and roots in the Cd400 treatment and Cd800 treatment groups was significantly greater than that in the CK treatment group. Compared with that in the Cd400 treatment group, the MDA content in the shoots in the Cd400 + Gly treatment group decreased significantly, by 39.86% and 25.88%, at 14 d and 21 d, respectively. Compared with that in the Cd800 treatment group, the MDA content in the shoots in the Cd400 + Gly treatment group decreased significantly, by 22.35% and 19.74%, at 14 d and 21 d, respectively. The application of exogenous glycerol also reduced the MDA content in the roots (Fig. 3b).

Under normal conditions, the application of exogenous glycerol had no significant effect on SOD activity in shoots or roots. Compared with that in the CK treatment, the SOD activity in the shoots and roots decreased significantly, and the SOD activity in the shoots was greater than that in the roots. At 14 d and 21 d of Cd stress, the SOD activity of shoots in the Cd400 + Gly treatment significantly increased by 14.13% and 34.29%, respectively, compared with that in the Cd400 treatment, and that in the Cd800 + Gly treatment significantly increased by 23.89% and 35.65%, respectively, compared with that in the Cd800 treatment (Fig. 3c).

At 14 d, compared with the POD activity of shoots and roots under CK treatment, that under Cd stress increased, but at 21 d, the POD activity under Cd stress decreased to different degrees compared with that under CK treatment, which may be due to the prolongation of Cd stress. At 14 d and 21 d, compared with that in the Cd400 treatment, the POD activity in the Cd400 + Gly treatment increased significantly, by 20.69% and 30.26%, respectively. The POD activity in the roots increased slightly. Compared with the Cd800 treatment, the Cd800 + Gly treatment significantly increased POD activity in the shoots, but POD activity in the roots did not increase significantly (Fig. 3d).

Exogenous glycerol slightly increased the CAT activity in the shoots and roots of plants not subjected to Cd stress. However, under Cd treatment, the CAT activity in the shoots and roots was significantly lower than that under CK treatment, and the greater the Cd concentration was, the more obvious the decrease in CAT activity was. Compared with the same concentration of Cd, the application of glycerol under Cd stress significantly increased the CAT activity in the shoots and increased the CAT activity in the roots, but these differences did not reach statistical significance (Fig. 3e).

### Cd content, cd transport coefficient and cd distribution in the subcellular fractions of maize seedlings

**Fig. 4:**
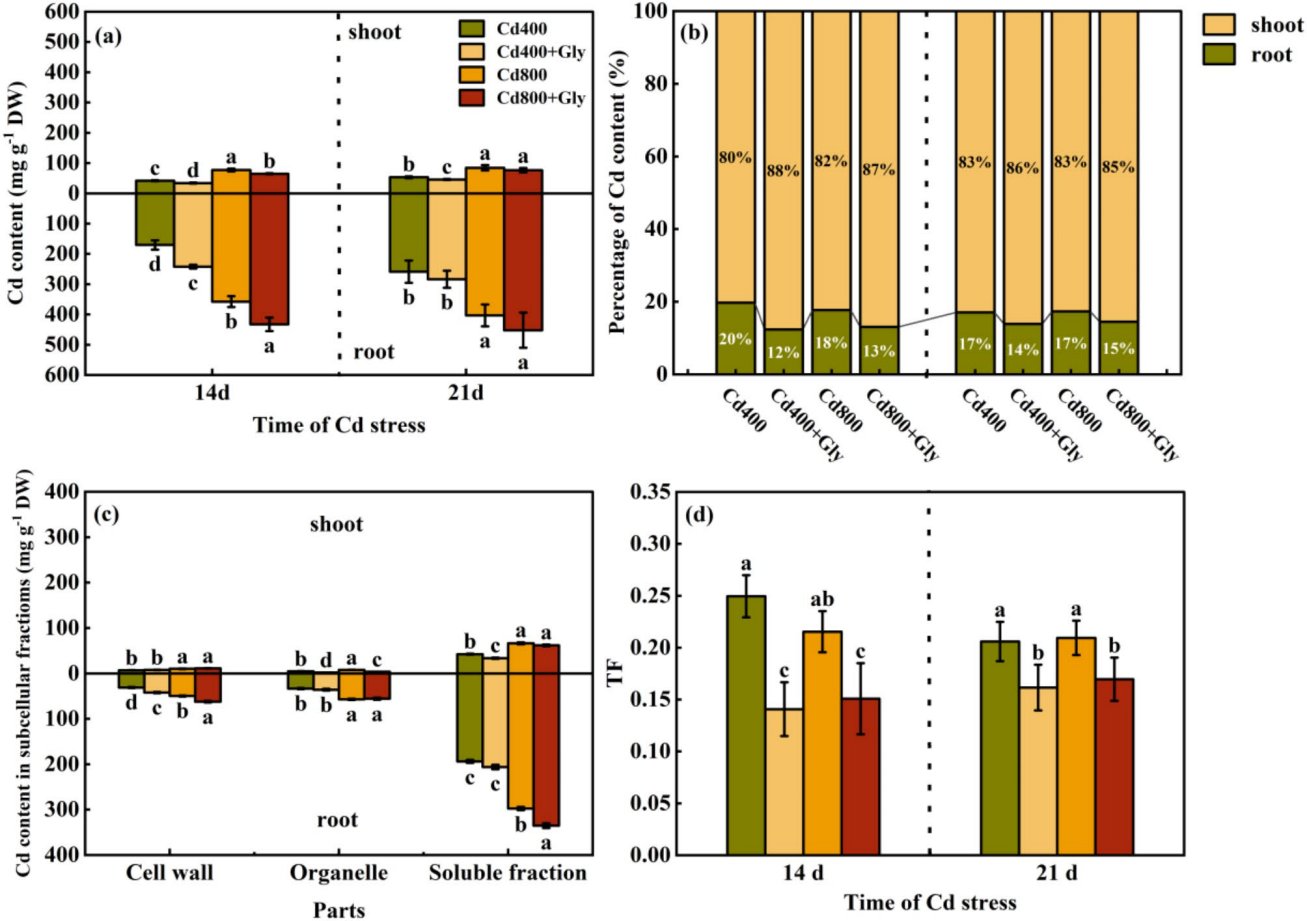
Effects of exogenous glycerol on the Cd content, Cd transport coefficient and Cd distribution in the subcellular fractions of maize under Cd stress. Different lowercase letters indicate that there are significant differences between different treatments at the same growth period at the level of *p* = 0.05, the same letter means no significant difference (*p* > 0.05), while different letters mean significant difference(*p* < 0.05). (**a**) Cd content of maize seedlings under different treatment conditions (mg g^-1^ DW), (**b**) Percentage of Cd content of maize seedlings under different treatment conditions (%), (**c**) Cd content in subcellular fractioms of maize seedlings under different treatment conditions (mg g^-1^ DW), (**d**) TF of maize seedlings under different treatment conditions. Data were presented as mean ± standard error of mean (SEM). One-way ANOVA. Post hoc Duncan’s analysis. Values of *p* < 0.05 were regarded as statistically significant.

Under Cd stress, Cd accumulated in the shoots and roots of maize, and the accumulation of Cd in the roots was greater than that in the shoots. Compared with that in the Cd400 treatment, the Cd content in the shoots in the Cd400 + Gly treatment was significantly lower (by 18.63% and 14.03%, respectively) at 14 d and 21 d, but the Cd content in the roots increased. Compared with that in the Cd800 treatment, the Cd content in the aboveground part in the Cd800 + Gly treatment decreased by 15.74% and 9.15% at 14 d and 21 d, respectively. The Cd content in the roots increased to a certain extent (Fig. 4a).

Figure 4b shows that at 14 d and 21 d, under Cd400 stress, the Cd content in the shoots after the application of glycerol accounted for 88% and 86%, respectively, of the total Cd content, which was 8% and 3% greater than the original Cd content. Moreover, under Cd800 stress, 86% and 85% of the total Cd content accumulated in the aboveground parts of the plants after the application of glycerol, corresponding to increases of 5% and 2%, respectively, compared with the original values.

In addition, under Cd stress, exogenous glycerol reduced the Cd transport coefficient. The TF in the Cd400 + Gly treatment was significantly lower than that in the Cd400 treatment at 14 d and 21 d (by 43.61% and 21.55%, respectively). Compared with that in the Cd800 treatment, the TF in the Cd800 + Gly treatment was significantly lower by 29.97% and 19.02% at 14 d and 21 d, respectively, indicating that exogenous glycerol could reduce the transfer of Cd to the aboveground parts of the plants (Fig. 4c).

Among the subcellular components of maize shoots and roots, soluble Cd was the main component. Moreover, the Cd content in maize root cells was much greater than that in aboveground cells. The Cd levels of organelles and soluble components in shoot cells in the Cd400 + Gly treatment group were significantly lower than those in the Cd400 treatment group (58.69% and 20.61%, respectively). Compared with the Cd800 treatment, the Cd800 + Gly treatment significantly decreased the Cd content by 53.82% and 7.91%, respectively. The Cd content in the cell wall increased slightly. In maize roots, compared with those in Cd400-treated plants, the Cd content in the cell wall in the Cd400 + Gly treatment increased significantly, by 35.83%, and the Cd content in organelles and soluble components was slightly greater than that in the Cd400 treatment group. Moreover, compared with that in the Cd800 treatment, the Cd content in the cell wall in the Cd800 + Gly treatment significantly increased by 25.20%, and the Cd content in the soluble components significantly increased by 12.65% (Fig. 4d).

### Transcriptome analysis

#### Quality evaluation of RNA-Seq sequencing data and differentially expressed genes

In this study, Illumina sequencing was used to complete the transcriptome sequencing of maize shoots after 21 days of treatment. Each treatment had three biological replicates, for a total of 18 samples, and quality control of the obtained original sequencing data was carried out. The sequence number of the raw data in the sequencing results was between 38,303,208 and 55,367,774, and the sequence number of the clean data after quality pruning was between 149,460,820 and 210,509,130. The base ratio of Q20 was ≥ 97.71%, and that of Q30 was ≥ 93.94%, indicating that the sequencing quality was high and could be analyzed in the next step (Table 1).

**Table 1:** Evaluation of the quality of the RNA-Seq data.

Sample	Raw reads	Clean reads	Error rate (%)	Q20 (%)	Q30 (%)
CK1	55,367,774	51,400,200	0.03	98.05	94.63
CK2	49,350,332	46,037,274	0.03	98.16	94.93
CK3	46,718,858	43,503,844	0.03	98.11	94.91
GL1	40,696,636	37,912,196	0.03	97.93	94.37
GL2	43,831,934	40,952,852	0.03	98.06	94.74
GL3	49,315,622	46,061,672	0.03	97.98	94.56
Cd4001	44,542,964	41,389,010	0.03	98.04	94.65
Cd4002	45,058,318	41,871,654	0.03	98.17	94.97
Cd4003	42,895,310	40,058,824	0.03	98.13	94.9
G4001	47,030,984	43,857,644	0.03	97.90	94.29
G4002	39,251,134	36,699,146	0.03	97.98	94.55
G4003	45,618,212	42,633,664	0.03	97.94	94.46
Cd8001	44,003,092	41,070,052	0.03	98.16	94.95
Cd8002	46,387,536	43,302,836	0.03	98.03	94.67
Cd8003	38,303,208	35,842,644	0.03	97.74	93.94
G8001	48,174,104	44,781,708	0.03	98.11	94.87
G8002	43,895,146	40,599,254	0.03	98.06	94.80
G8003	50,392,116	46,806,988	0.03	98.17	95.09

CK1, CK2, and CK3 represent the three repeats of the control treatment; GL1, GL2, and GL3 represent the triplicates of the Gly treatment; Cd4001, Cd4002, and Cd4003 represent the three repeats of the Cd400 treatment; G4001, G4002, and G4003 represent the three repeats of the Cd400 + Gly treatment; Cd8001, Cd8002, and Cd8003 represent the three repeats of the Cd800 treatment; and G8001, G8002, and G8003 represent the three repeats of the Cd800 + Gly treatment. Statistical table of data output of each sample.

#### GO function annotation analysis and KEGG enrichment analysis of differentially expressed genes

**Fig. 5:**
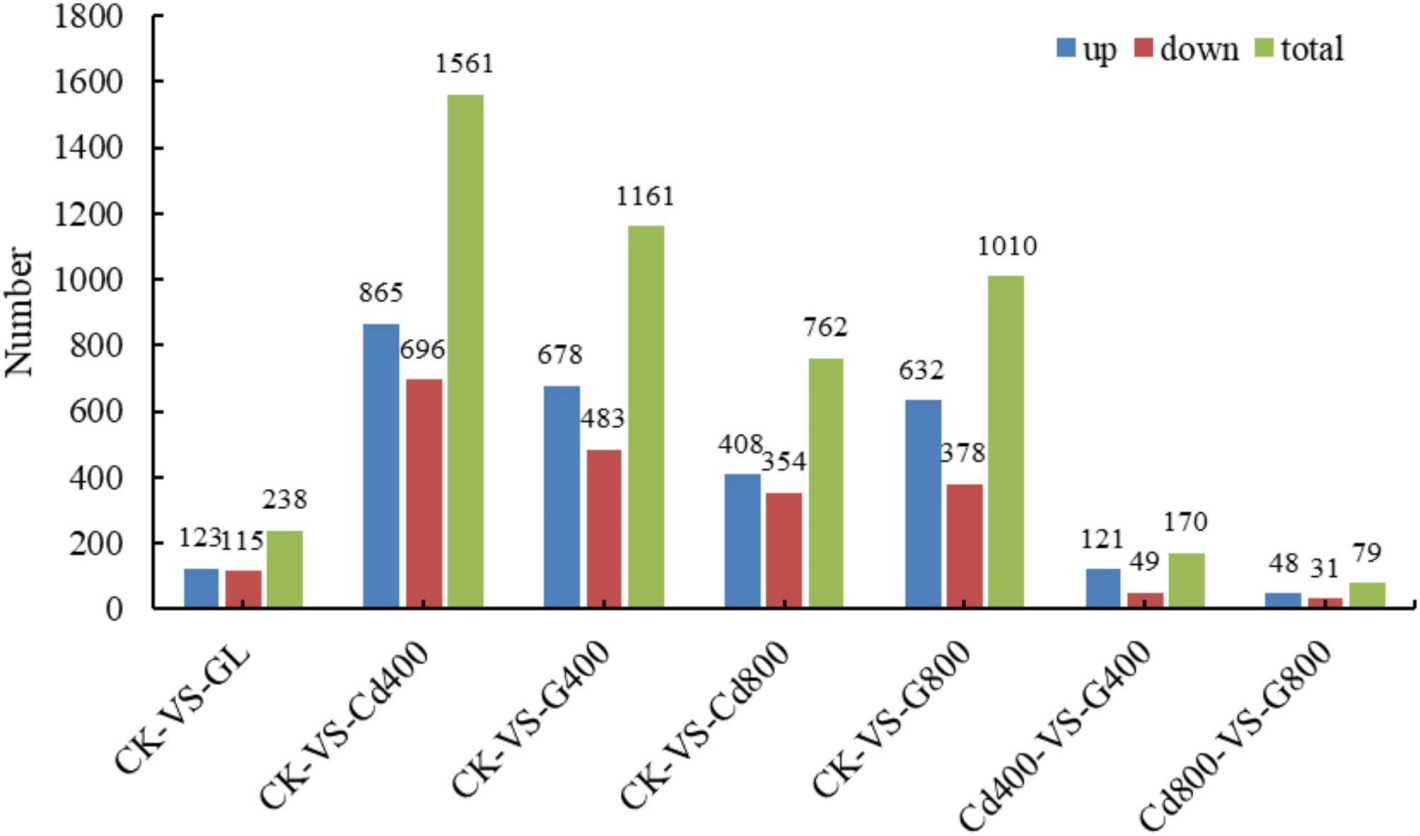
The number of differentially expressed genes among the different treatment groups. CK represents the control group; GL represents the Gly treatment; Cd400 represents the Cd400 treatment; G400 and Cd800 represent the Cd800 treatment; and G800 represents the Cd800 + Gly treatment.

The number of differentially expressed genes between the different treatment groups is shown in Fig. 5. There were 238 differentially expressed genes between the CK and Gly treatment groups, including 123 upregulated genes and 115 downregulated genes. A total of 1561 differentially expressed genes were obtained between the CK and Cd400 treatments, and 865 upregulated genes were more common than 696 downregulated genes. A total of 1161 differentially expressed genes were detected in the Cd400 + Gly treatment group compared with the Cd400 + Gly treatment group, of which 678 were upregulated and 483 were downregulated. A total of 762 differentially expressed genes were identified between the Cd800 treatment group and the control group, and 408 and 354 genes were upregulated and downregulated, respectively. There were 1010 differentially expressed genes between the CK and Cd800 + Gly treatment groups, and the numbers of upregulated genes and downregulated genes were 632 and 378, respectively. There were 170 differentially expressed genes, 121 upregulated genes and 49 downregulated genes in the Cd400 + Gly treatment group compared with those in the Cd400 treatment group. A total of 79 differentially expressed genes were detected between the Cd800 treatment group and the Cd800 + Gly treatment group, 48 of which were upregulated and 31 of which were downregulated.

**Fig. 6:**
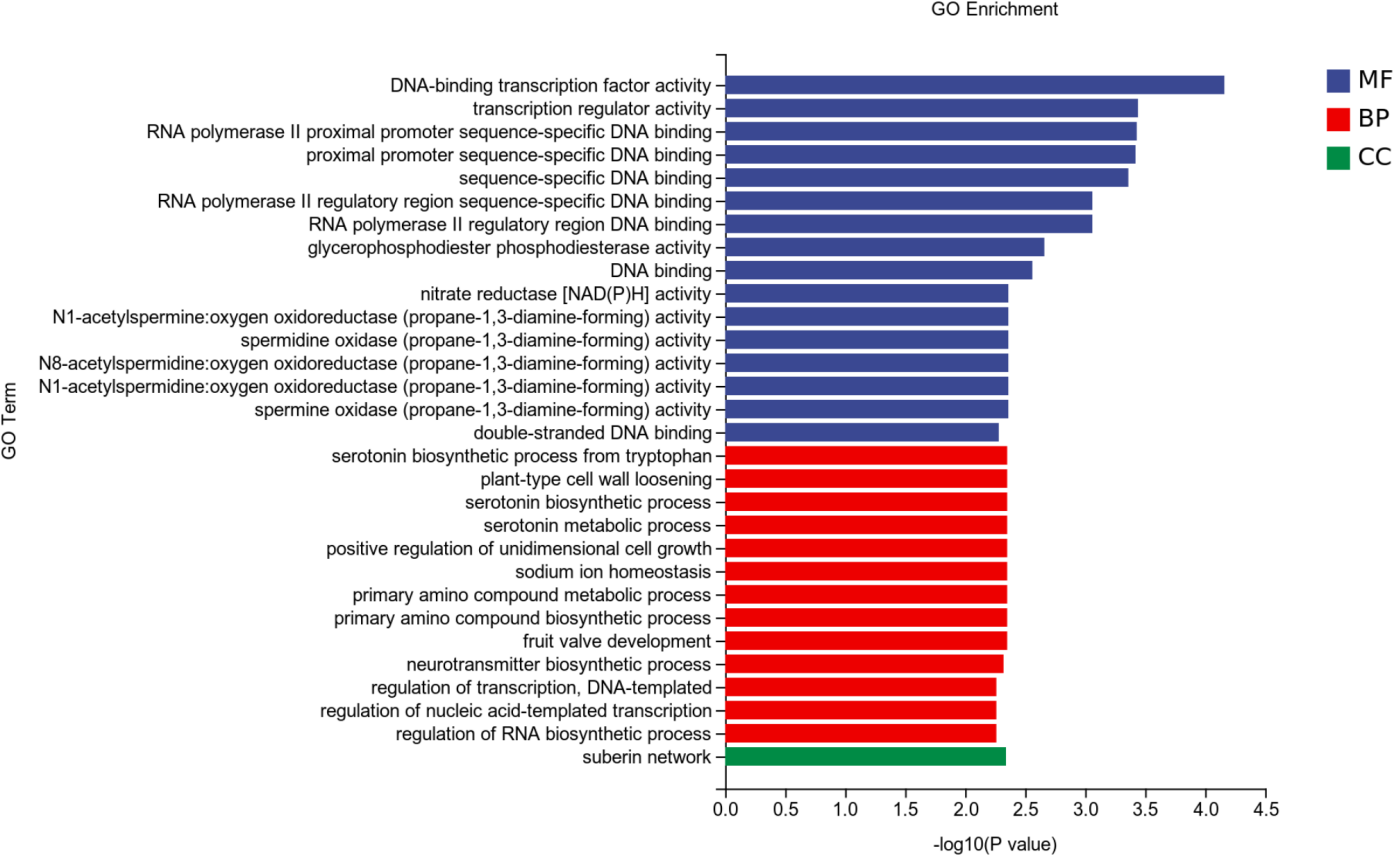
Histogram of GO enrichment analysis data for differentially expressed genes in the Cd400 and Cd400 + Gly treatment groups.

**Fig. 7:**
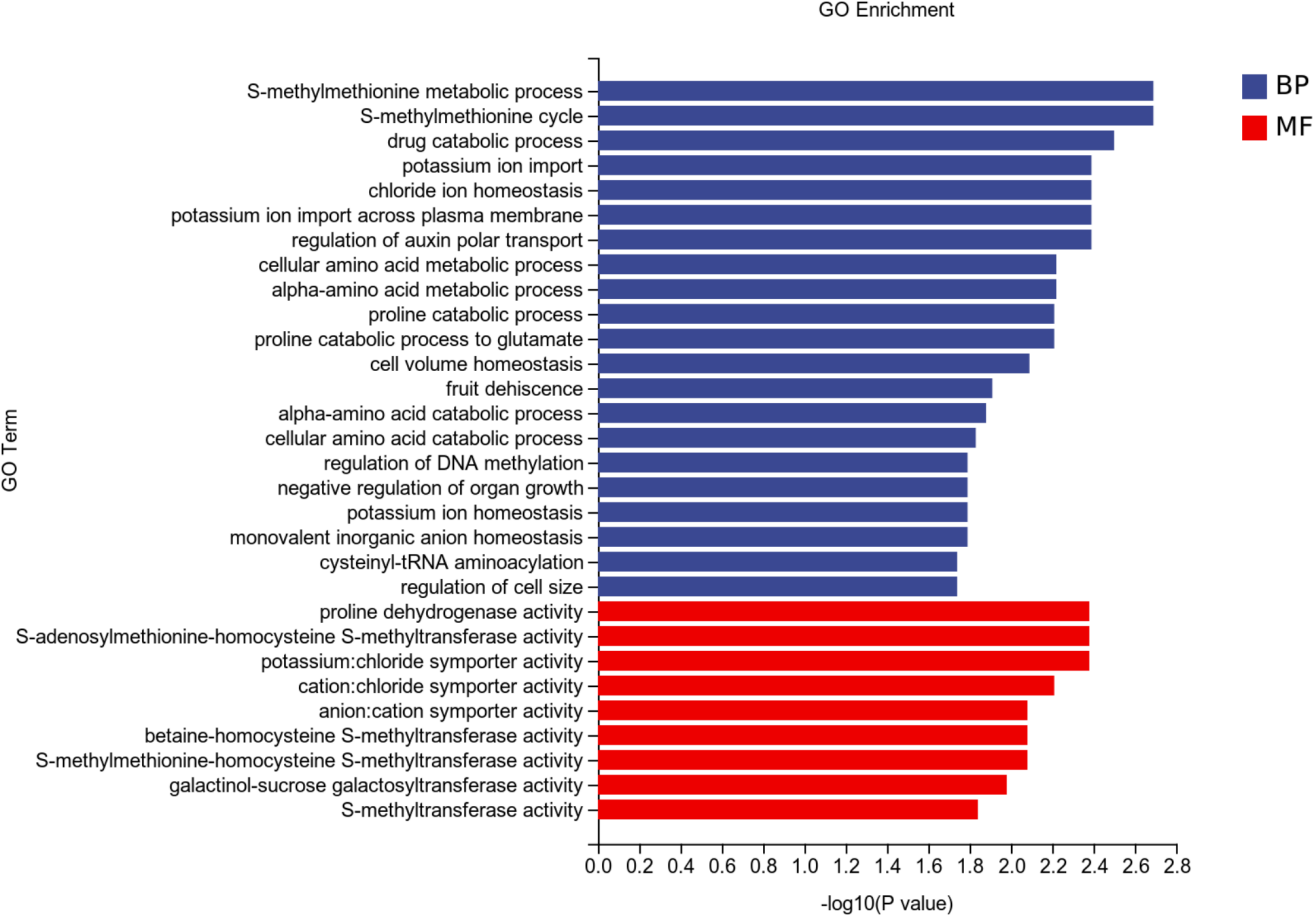
Histogram of GO enrichment analysis data for differentially expressed genes in the Cd800 and Cd800 + Gly treatment groups.

The GO terms associated with the differentially expressed genes in the Cd400/Cd400 + Gly treatment group were enriched mainly in the first-level terms related to molecular function and biological process. The secondary enriched terms were DNA-binding transcription factor activity, transcription regulator activity, glycerophosphodiester phosphodiesterase activity, nitrate reductase [NAD(P) H] activity, positive regulation of unidimensional cell growth sodium ion homeostasis, and primary amino compound metabolic process (Fig. 6). The Cd800/Cd800 + Gly treatment group was enriched mainly in S-methylmethionine metabolic process, S-methylmethionine cycle, drug catabolic process, chloride ion homeostasis, potassium ion import across the plasma membrane, regulation of polar auxin transport, cellular amino acid metabolic process, proline catabolic process, proline catabolic process to glutamate, cell volume homeostasis, anion: cation symporter activity, and galactinol-sucrose galactosyltransferase activity (Fig. 7).

**Fig. 8:**
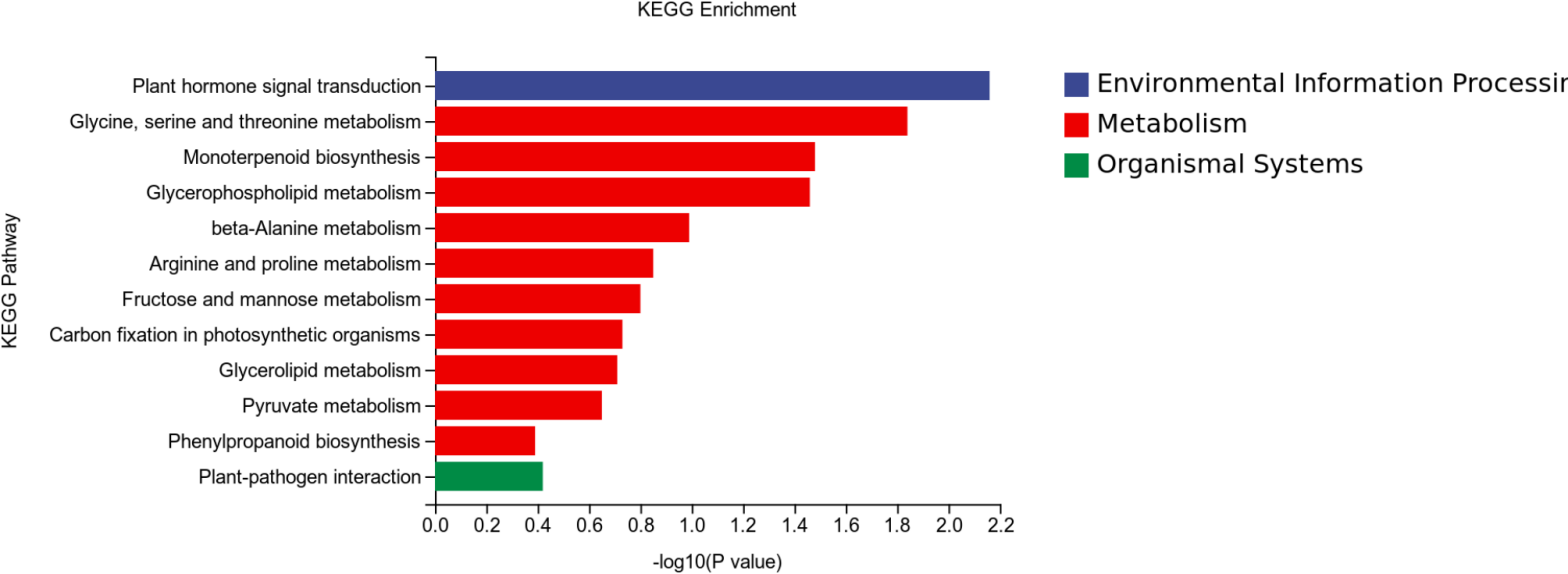
Histogram of KEGG enrichment data for DEGs between the Cd400 and Cd400 + Gly treatment groups.

**Fig. 9:**
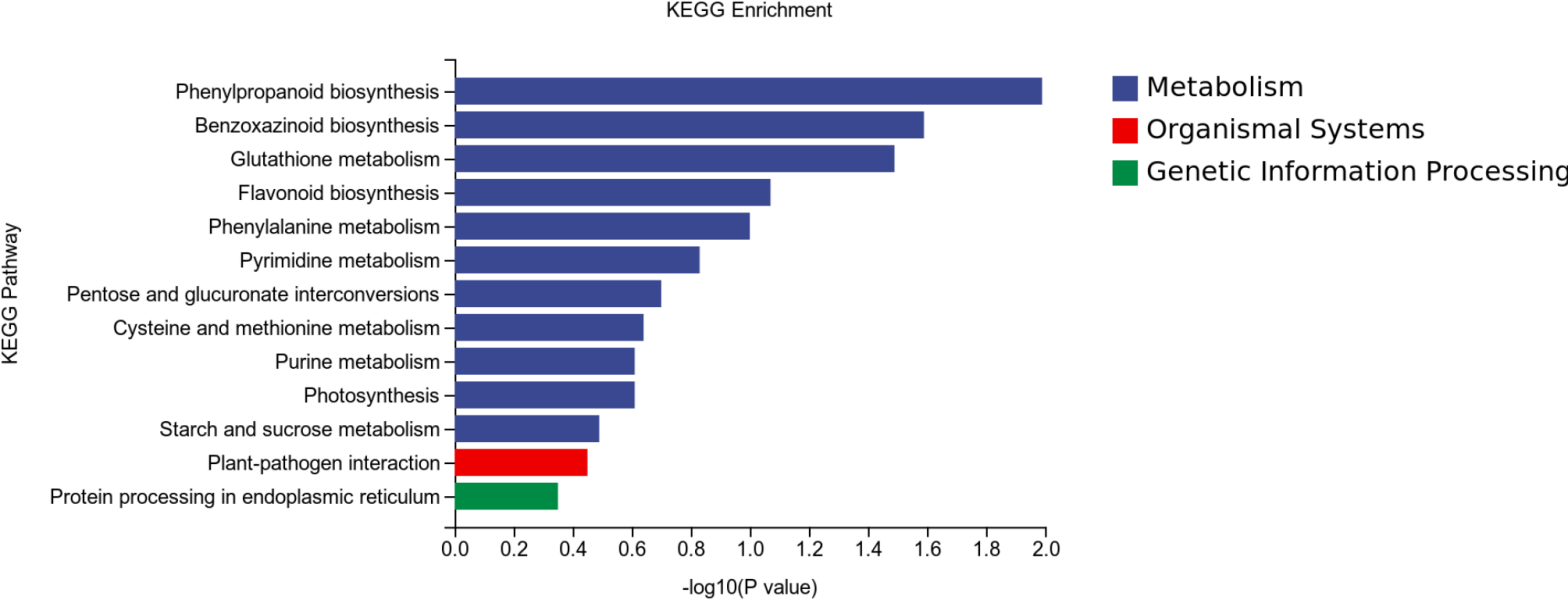
Histogram of KEGG enrichment data for DEGs between the Cd800 and Cd800 + Gly treatment groups.

The differentially expressed genes identified via KEGG pathway analysis between the Cd400/Cd400 + Gly treatment groups were enriched in 12 pathways, including plant hormone signal transduction; glycine, serine and threonine metabolism; monoterpenoid biosynthesis; glycerophospholipid metabolism; beta-alanine metabolism; arginine and proline metabolism; fructose and mannose metabolism; carbon fixation in photosynthetic organisms; glycerolipid metabolism; pyruvate metabolism; phenylpropanoid biosynthesis; and plant‒pathogen interactions (Fig. 8). The Cd800/Cd800 + Gly treatment group was analyzed. The enriched pathways included phenylpropanoid biosynthesis, benzoxazinoid biosynthesis, glutathione metabolism, flavonoid biosynthesis, phenylalanine metabolism, pyrimidine metabolism, pentose and glucuronate interconversions, cysteine and methionine metabolism, purine metabolism, photosynthesis, starch and sucrose metabolism, plant‒pathogen interactions, and protein processing in the endoplasmic reticulum (Fig. 9).

## Discussion

Cd can cause significant damage to maize plants, thereby reducing the nutrient and water supplies from roots to shoots^22^. However, the damage caused by Cd to plants can be reduced by spraying plants with exogenous substances on leaves^23,24^. Previous studies have shown that glycerol is an important carbon source for tissue culture and can support cell growth, and foliar spraying of glycerol can promote the growth of healthy plants^25^. This study revealed that foliar spraying of glycerol promoted the growth of maize seedlings under Cd stress and significantly increased shoot and root dry weights and fresh weights while significantly increasing shoot height, total length and the length of the roots on the root surface (Fig. 1, Figure S1 a and b). However, it had little effect on the root volume or average root diameter (Figure S1 c and d). This could be attributed to the absorption of glycerol by leaves as a foliar fertilizer, which affects photosynthesis and carbon and nitrogen metabolism in maize. This process is beneficial for the accumulation of dry matter above ground. In addition, it is possible that Cd directly damages roots and indirectly damages aboveground parts. However, glycerol directly acts on the leaf surface and is absorbed and utilized by the leaf surface to promote the growth of the aboveground parts. Therefore, the mitigation effect on the aboveground parts is greater than that on the belowground parts, which can effectively alleviate the continuous indirect harm caused by Cd.

The growth and development of plants and the accumulation of organic matter are inseparable from plant photosynthesis and are the main indices used to evaluate plant damage caused by abiotic stress^26,27^. Under adverse conditions, the photosynthetic system of crops is damaged, which strongly affects the carbon assimilation process^28,29^. Many studies have focused on alleviating the damage caused by Cd to plants through different measures and subsequently maintaining the increased photosynthetic assimilation ability of plants^30^. For example, through the combined application of silicon and nitric oxide, the damage caused by Cd stress to maize can be alleviated, the chlorophyll content and photosynthetic rate of leaves can increase, and ultimately, the yield can increase^31^. In addition, rice plants protect against photosynthesis inhibition by increasing nitrogen assimilation via the action of exogenous L-glutamic acid^32^. This finding is similar to our results. In the present study, the application of glycerol under Cd stress increased the photosynthetic capacity of the maize seedlings. The total chlorophyll content, *Pn*, Stomatal conductance (*Gs*), and Transpiration rate (*Tr*) all increased to varying degrees, but Intercellular CO₂ concentration (*Ci*) decreased. (Fig. 2a and b, Figure S2 a, b and c). These findings indicated that the ability of cells to fix and assimilate CO_2_ was enhanced and that the photosynthetic mechanism of maize shoots under Cd stress was improved. In the present study, *RuBisCO* activity and *PEPC* activity in maize shoots increased (Fig. 2c and d). Zhou^33^ reported that the abundance of *RuBisCO* and *PEPC* in maize leaves decreased significantly under Cd stress and that the abundance of *RuBisCO* and *PEPC* in leaves increased significantly after the addition of Ca to the nutrient solution. Transcriptomic evidence further supports these physiological improvements, which revealed that glycerol treatment enriched pathways related to phenylpropanoid biosynthesis and photosynthesis (Figs. 8 and 9). The upregulation of these pathways suggests that glycerol enhances carbon fixation and secondary metabolite synthesis, collectively mitigating Cd-induced oxidative damage (Fig. 3). In addition, the photosynthetic performance index (*PIabs*) and Maximum quantum yield of PSII (*Fv/Fm*) can more accurately reflect the damage caused by plant light cooperation institutions^34^. The results of this study showed that the application of exogenous glycerol under Cd stress could reduce the decrease in PIabs; however, the effect on the Fv/Fm ratio was not significant. (Figure S2 d and e). These findings suggest that glycerol enhances carbon assimilation and light utilization capacity. According to this theory, an increase in the *Pn* and key photosynthesis-related enzyme activity affects the amount of photosynthetic assimilation products. This study revealed that the application of exogenous glycerol under Cd stress significantly increased the sucrose and total carbohydrate levels in maize shoots (Fig. 2e and f). Taken together, glycerol treatment can increase corn biomass (Fig. 1), suggesting that glycerol may act as a carbon skeleton to support metabolic processes under stress. By using exogenous glycerol, plants may form an intermediate product of the Calvin cycle, which provides a carbon skeleton for the carbon assimilation process. This improves the photosynthetic performance of maize plants and the activity of key photosynthesis-related enzymes and increases the accumulation of photosynthetic assimilation products. This study lays a good foundation for the formation of final biomass.

Abiotic stress can lead to the rapid production of a large amount of Reactive Oxygen Species (ROS) in plants, damaging cells and ultimately leading to membrane lipid peroxidation^35^. Long-term Cd stress causes damage to maize tissues, thereby increasing the toxicity of Cd to maize seedlings and reducing their antioxidant capacity^36,37^. Fortunately, antioxidant systems in plants, such as those that use antioxidant enzymes, remove intracellular ROS and play a defensive role^38^. Higher SOD and POD activities are beneficial for removing intracellular ROS, whereas CAT and Ascorbate peroxidase (APX) are the main enzymes involved in removing H_2_O_2_ under Cd stress by catalyzing the decomposition of H_2_O_2_ into water and oxygen^39^. Exogenous proline alleviates Cd stress by promoting the activities of SOD, POD and Glutathione peroxidase (GPX) in the leaves and roots of young jujube trees^40^. As in the present study, the activities of various antioxidant enzymes in the aboveground parts significantly increased. However, some enzyme activity in the roots was not significantly induced, which indicated that glycerol resisted Cd damage mainly by activating the protective enzyme system in the leaves (Fig. 3c, d and e, Figure S3 a). Related studies have shown that under Cd stress, the content of nonenzymatic antioxidants such as Ascorbic Acid (AsA) and Glutathione (GSH) decreases^41^. However, under the action of exogenous 6-BA, the levels of AsA and GSH increase under stress^42^. Research shows that under Cd stress, the ability of rape to scavenge peroxides is greatly improved by the presence of exogenous melatonin, which reduces H_2_O_2_ and MDA contents, decreases membrane lipid peroxidation, and enhances antioxidant defense^43^. This finding is consistent with the results of the present study. As in the present study, the antioxidant system was disrupted under Cd stress, leading to a significant reduction in the activity of nonenzymatic antioxidants. However, after the application of glycerol, the levels of AsA and GSH increased significantly or even returned to their initial levels, and the antioxidant defense ability improved (Figure S3 b, d). Moreover, the H_2_O_2_ and MDA levels in maize leaves and roots increased significantly under Cd stress (Fig. 3a and b), which caused great damage to the cell membrane, resulting in a significant increase in the electrolyte leakage (EL) rate (Figure S3 c). However, the application of glycerol can could reduce the degree of membrane lipid peroxidation in cells so that H_2_O_2_, MDA and EL decrease, indicating that glycerol can alleviate the damage caused by Cd in cells. The transcriptomic data further revealed that the glycerol treatment enriched pathways related to flavonoid biosynthesis (Fig. 9), which are known to enhance antioxidant defense mechanisms. These findings suggest that glycerol not only enhances the activity of antioxidant enzymes but also modulates the expression of genes involved in ROS scavenging, thereby reducing oxidative stress and improving plant tolerance to Cd. Therefore, glycerol may act as an activator of antioxidant enzyme activity by increasing the activity of antioxidant enzymes and enhancing the protective mechanism of the antioxidant system while reducing peroxides to respond to the physiological regulation of maize under stress.

The longer the duration of Cd stress is, the more Cd accumulates in plants, and more Cd-induced damage occurs in plants^44,45^. Previous studies have demonstrated that under the combined influence of nanoparticles and melatonin, wheat can reduce the absorption of Cd, reduce the accumulation of Cd in plant organs, and improve the tolerance of Cd^21^. In addition, the transport of absorbed Cd in crops is critical. For example, in rice, Cd absorbed by roots is transferred to shoots, and the Cd accumulated in leaves directly affects the Cd content of infinal brown rice^46^. The results of this experiment are basically consistent with the above experimental results. The Cd content in the roots of the maize seedlings in this study was much greater than that in the shoots, and exogenous glycerol significantly reduced the Cd content in the shoots (Fig. 4a). However, the Cd content in the roots significantly increased. In addition, compared with Cd stress, the application of glycerol significantly reduced TF levels (Fig. 4d). Although glycerol did not effectively reduce Cd uptake or accumulation in maize seedlings, it reduced Cd toxicity, prevented Cd transfer from belowground to aboveground parts, and decreased the Cd content in leaves and other tissues.Therefore, the regulatory pattern by which glycerol affects Cd transport in maize seedlings may involve influencing long-distance transport from roots to leaves, reducing the risk of Cd accumulation in mature grains. After glycerol was sprayed on the rice leaves, the amount of Cd in the soluble components of the leaf cells decreased, and the vacuoles in the leaf cells were enriched with Cd. Moreover, soluble Cd and inorganic Cd chelated with pectin accumulate in the cell wall, detoxify Cd, and reduce the transfer of Cd to other tissues and cells of crops, thereby reducing Cd toxicity^14^. In addition, salicylic acid can induce the synthesis of polysaccharides in the tomato cell wall, through which Cd is chelated and fixed^47^. In the present study, the Cd content in the soluble fraction of plant leaves was greater than that in the cell wall and organelles under Cd treatment. However, foliar application of glycerol reduced the Cd content in the organelles and soluble fractions while slightly increasing it in the cell wall. In the subcellular fractions of maize roots, glycerol treatment increased the content of Cd in both the cell wall and soluble fractions (Fig. 4c). Transcriptomic analysis revealed that glycerol treatment enriched pathways related to phenylpropanoid biosynthesis and plant-pathogen interactions (Figs. 8 and 9), which are known to increase cell wall integrity and reduce Cd mobility. These findings indicate that exogenous glycerol may be utilized by maize to provide a carbon skeleton for polysaccharide synthesis within the cell wall. It can also chelate Cd, fix it within the root cell wall, reduce its transport to aboveground parts, and decrease its content within leaf organelles and soluble components. This reduces the damage caused by Cd stress in plants. As an initial line of defense, root systems play a crucial role in enhancing resistance against Cd stress in maize seedlings.

To further elucidate on the molecular basis of glycerol-mediated alleviation of Cd stress, transcriptomic analysis was conducted^48–50^. The physiological data revealed that glycerol significantly improved the antioxidant capacity, photosynthetic efficiency, and Cd sequestration in maize seedlings (Figs. 1, 2, 3 and 4). Transcriptomic analysis provided deeper insights into the underlying molecular patterns associated with these physiological improvements^51^. The differentially expressed genes in the CK/Cd400 treatment group and CK/Cd800 treatment group were enriched mainly in photosynthesis, photosystem, redox process, chloroplast and other terms related to photosynthesis (Figures S4 and S5). This finding corroborates the observed decline in chlorophyll content and *RuBisCO*/*PEPC* activity under Cd stress (Fig. 2a, c, and d), suggesting that Cd directly impairs the photosynthetic machinery at both the molecular and physiological levels. Notably, glycerol treatment upregulated photosynthesis-related pathways (Fig. 6), which may explain the restored photosynthetic rates and increased carbohydrate levels in glycerol-treated plants (Fig. 2). These findings indicate that glycerol mitigates Cd-induced photosynthetic inhibition by enhancing carbon fixation and energy metabolism. In the Cd400/Cd400 + Gly treatment group, DEGs were enriched in pathways related to glycerophosphodiesterase activity, nitrate reductase activity, and sodium ion homeostasis (Fig. 6). These pathways are critical for nitrogen assimilation and ion balance, which are often disrupted under Cd stress. The upregulation of these pathways by glycerol likely contributes to the improved nutrient uptake and biomass accumulation observed in glycerol-treated plants (Figs. 1 and 2e-f). These findings suggest that glycerol not only alleviates Cd toxicity but also enhances nutrient utilization efficiency, thereby promoting plant growth under stress conditions. In the Cd800/Cd800 + Gly treatment group, the differentially expressed genes were enriched mainly in the S-methylmethionine metabolic process, chloride ion homeostasis, regulation of auxin polar transport, the cellular amino acid metabolic process, and galactosyl sucrose galactosyltransferase activity (Fig. 7), which could explain the improved root morphology and biomass accumulation (Fig. 1b and c). The enrichment of phenylpropanoid biosynthesis pathways in transcriptomic data (the Cd400/Cd400 + Gly and Cd800/Cd800 + Gly treatment groups) aligns with the observed increase in Cd sequestration within the root cell walls (Figs. 4d, 8 and 9), suggesting that glycerol enhances cell wall integrity to immobilize Cd. This molecular pattern likely contributes to the reduced TF and shoot Cd content (Fig. 4a-c), highlighting glycerol’s dual role in enhancing antioxidant defense and immobilizing Cd in roots. The KEGG enrichment pathway of differentially expressed genes between the CK group, the 400 µM and 800 µM Cd treatment groups revealed that Cd stress affects photosynthesis, (Figure S6 and S7), which is consistent with the elevated H_2_O_2_ and MDA levels (Fig. 3a and b). However, glycerol supplementation counteracted these effects by upregulating photosynthesis-related pathways and phenylpropanoid biosynthesis (Figs. 8 and 9). Moreover, the physiological data revealed increases increase in SOD, POD and CAT activities, and decreases in H_2_O_2_ and MDA levels (Fig. 3). The upregulation of these pathways suggests that glycerol enhances the antioxidant capacity of maize seedlings, thereby mitigating Cd-induced oxidative stress. These transcriptomic findings provide a molecular basis for physiological improvements, including restored photosynthetic rates, reduced oxidative damage, and suppressed Cd translocation. This may be related to phenylpropanoid biosynthesis, plant‒pathogen interactions and photosynthesis pathways, and the molecular mechanism of specific mitigation needs to be further studied. Future studies should focus on validating key genes (e.g., those involved in phenylpropanoid biosynthesis and photosynthesis). Additionally, the interplay between glycerol-mediated gene regulation and physiological responses warrants further investigation to fully elucidate the complex mechanisms of Cd stress mitigation.

## Electronic supplementary material

Below is the link to the electronic supplementary material.

Supplementary Material 1

## Data Availability

The reads produced in this study have been deposited in the National Center for Biotechnology Information (NCBI) SRA database under the accession number SRP 520234. Access to the data are available upon publication at https://www.ncbi.nlm.nih.gov/sra/?term=SRP520234Under reasonable requirements, the physiological datasets generated in this study can be obtained from the corresponding authors.
