# Supplementary material for: Physiological regulation underlying the alleviation of cadmium stress in maize seedlings by exogenous glycerol
Source: Sci Rep. 2025 Apr 1;15:11156. doi: 10.1038/s41598-025-94385-4 (PMC11961616; doi:10.1038/s41598-025-94385-4)

**Figure Supplement Captions:**

**Figure** **S1** Effects of exogenous glycerol on morphological indexes of maize seedlings under Cd stress. Different lowercase letters indicate that there are significant differences between different treatments at the same growth period at the level of p=0.05, the same letter means no significant difference (p>0.05), while different letters mean significant difference(p<0.05). (a) Fresh weight of shoot and root of maize seedlings under different treatment conditions (g plant^-1^), (b) Shoot height of maize seedlings under different treatment conditions (cm), (c) Root volume of maize seedlings under different treatment conditions (cm^3^), (d) Average root of maize seedlings under different treatment conditions (mm). Data were presented as mean ±standard error of mean (SEM). One-way ANOVA. Post hoc Duncan’s analysis. Values of p<0.05 were regarded as statistically significant.

**Figure S2** Effects of exogenous glycerol on photosynthetic performance of maize seedlings under cadmium stress. Different lowercase letters indicate that there are significant differences between different treatments at the same growth period at the level of p=0.05, the same letter means no significant difference (p>0.05), while different letters mean significant difference(p<0.05). (a) *Ci* of maize seedlings under different treatment conditions (μmol mol^-1^）, (b) *Gs* of maize seedlings under different treatment conditions （mmol m^-2^s^-1^）, (c) *Tr* of maize seedlings under different treatment conditions (mmol m^-2^s^-1^), (d) Fv/Fm of maize seedlings under different treatment conditions, (e) *Plabs* of maize seedlings under different treatment conditions. Data were presented as mean ±standard error of mean (SEM). One-way ANOVA. Post hoc Duncan’s analysis. Values of p<0.05 were regarded as statistically significant.

**Figure** **S3** Effects of exogenous glycerol on antioxidant system of maize seedlings under Cd stress. Different lowercase letters indicate that there are significant differences between different treatments at the same growth period at the level of p=0.05, the same letter means no significant difference (p>0.05), while different letters mean significant difference (p<0.05).

**Figure S4** Histogram of GO enrichment analysis of differentially expressed genes in CK and Cd 400 treatment groups.

**Figure S5** Histogram of GO enrichment analysis of differentially expressed genes in CK and Cd 800 treatment groups.

**Figure S6** Histogram of KEGG enrichment of differential gene between CK and Cd 400 treatment groups.

**Figure S7** Histogram of KEGG enrichment of differential gene between CK and Cd 800 treatment groups.

**Figure S 1**


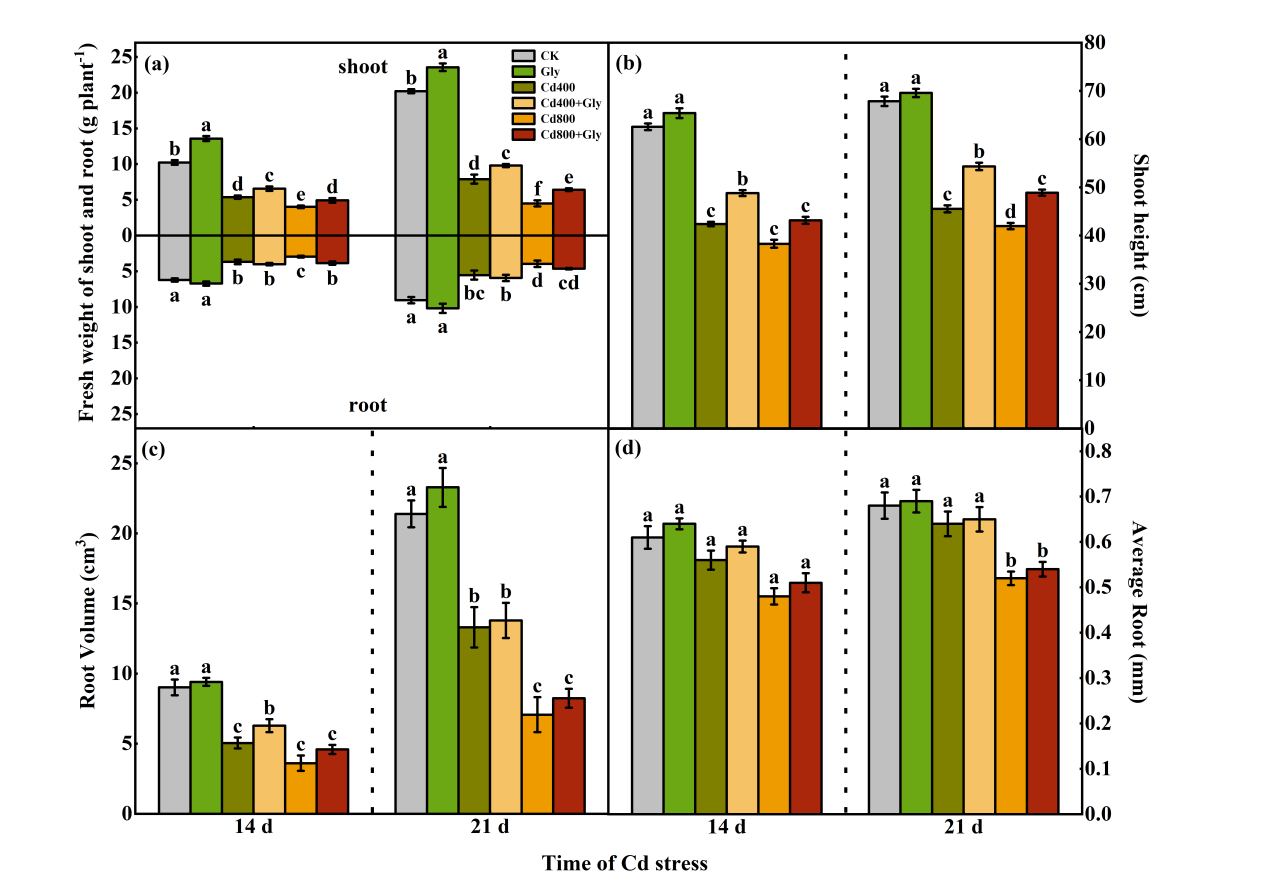


**Figure S 2**

**
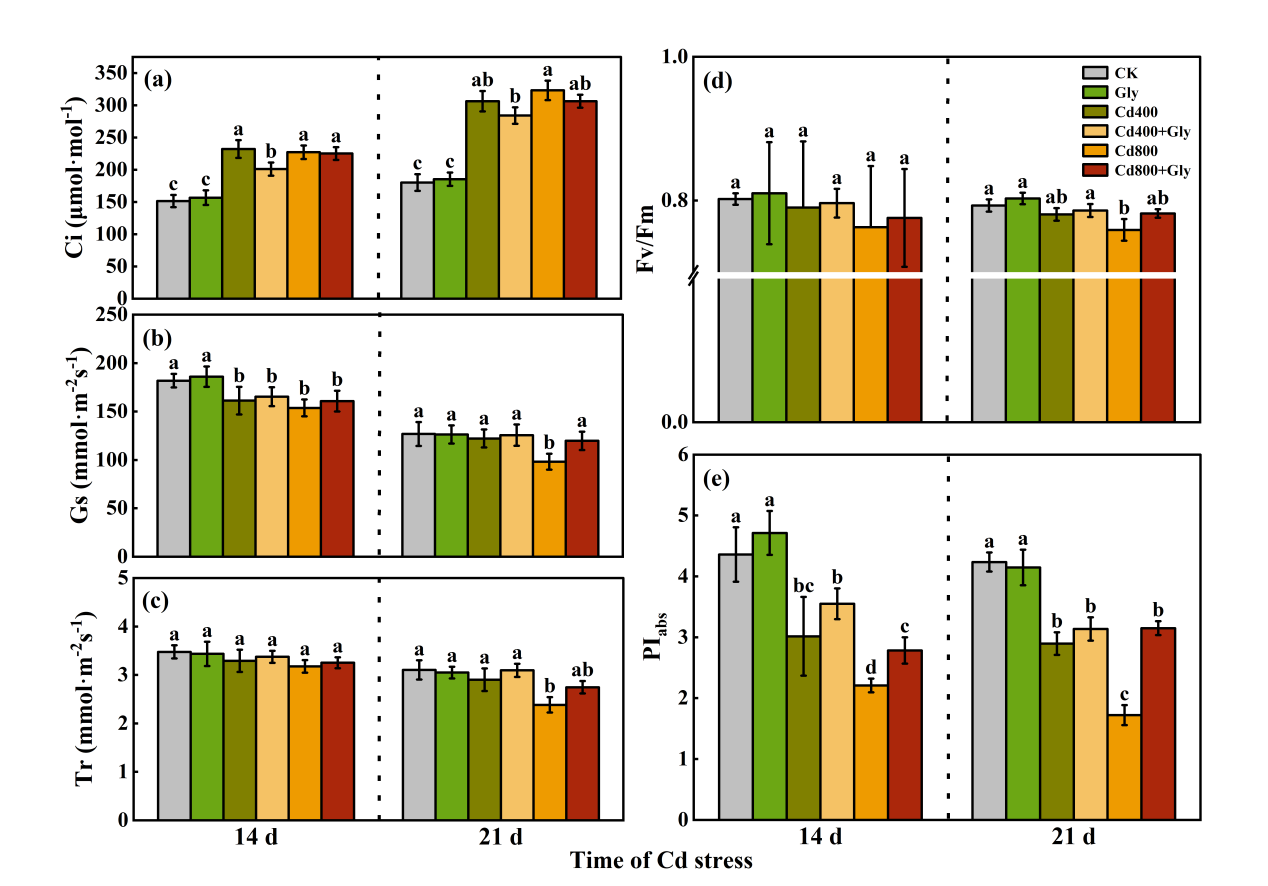
**

**Figure S 3**

**
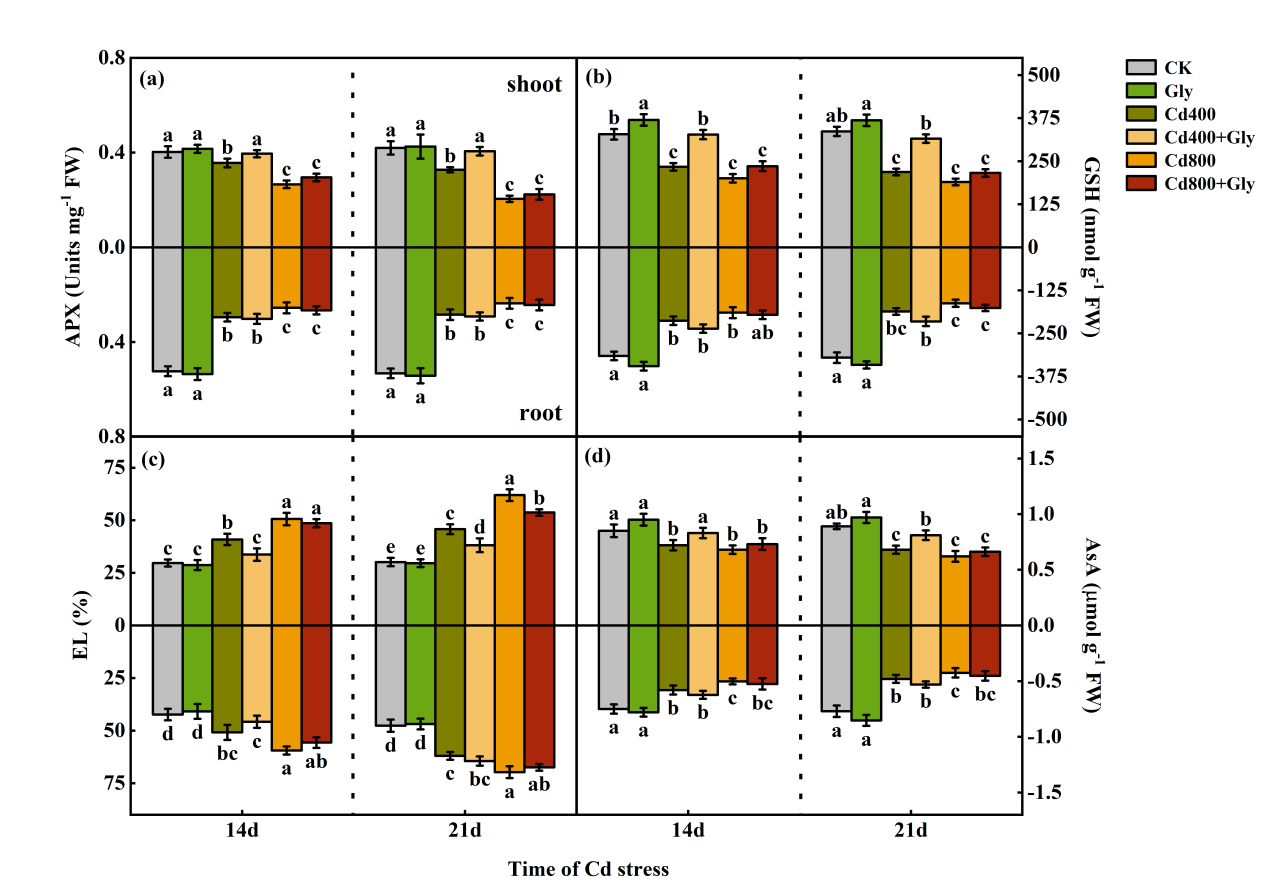
**

**Figure S 4**


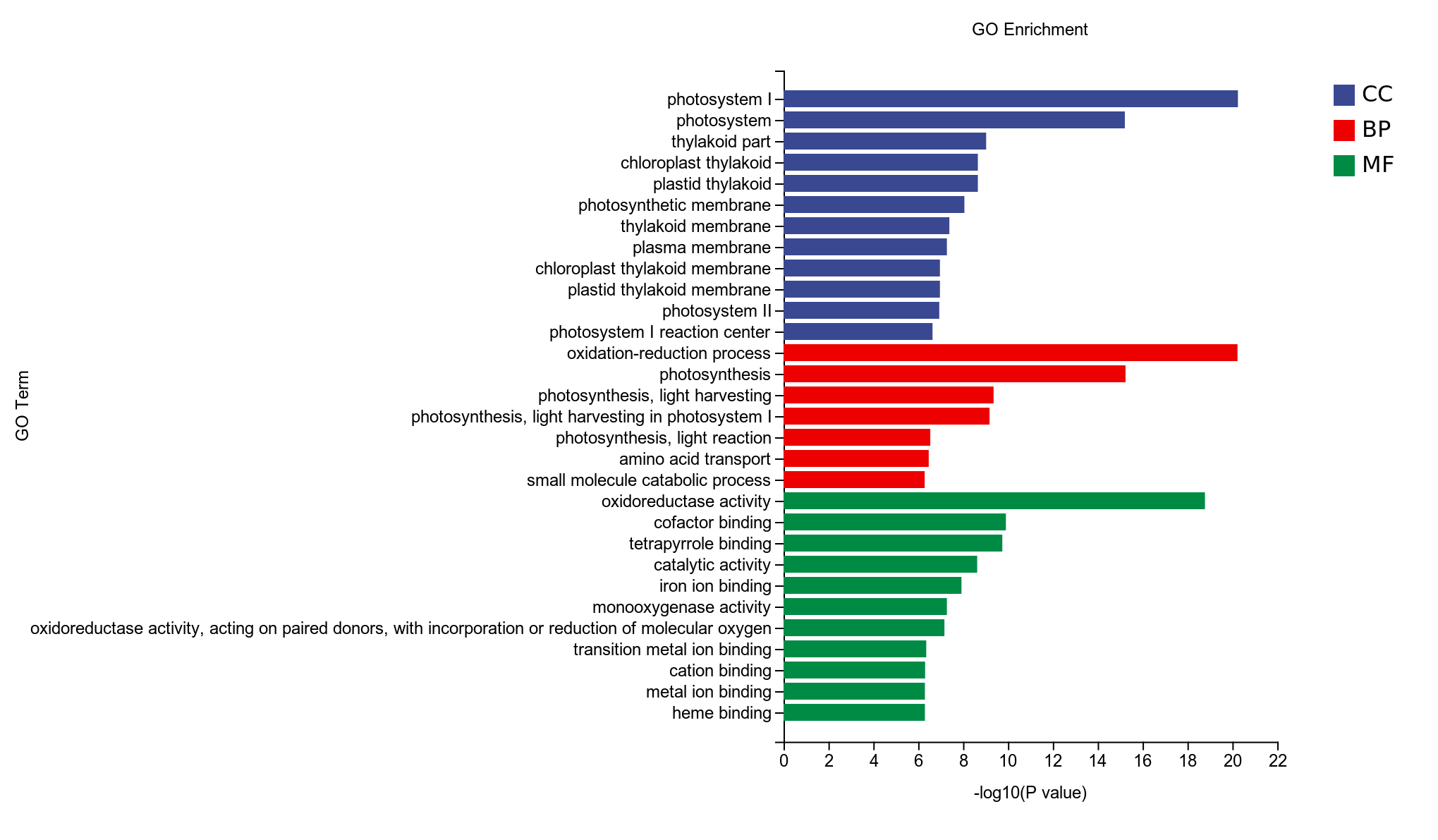


**Figure S 5**


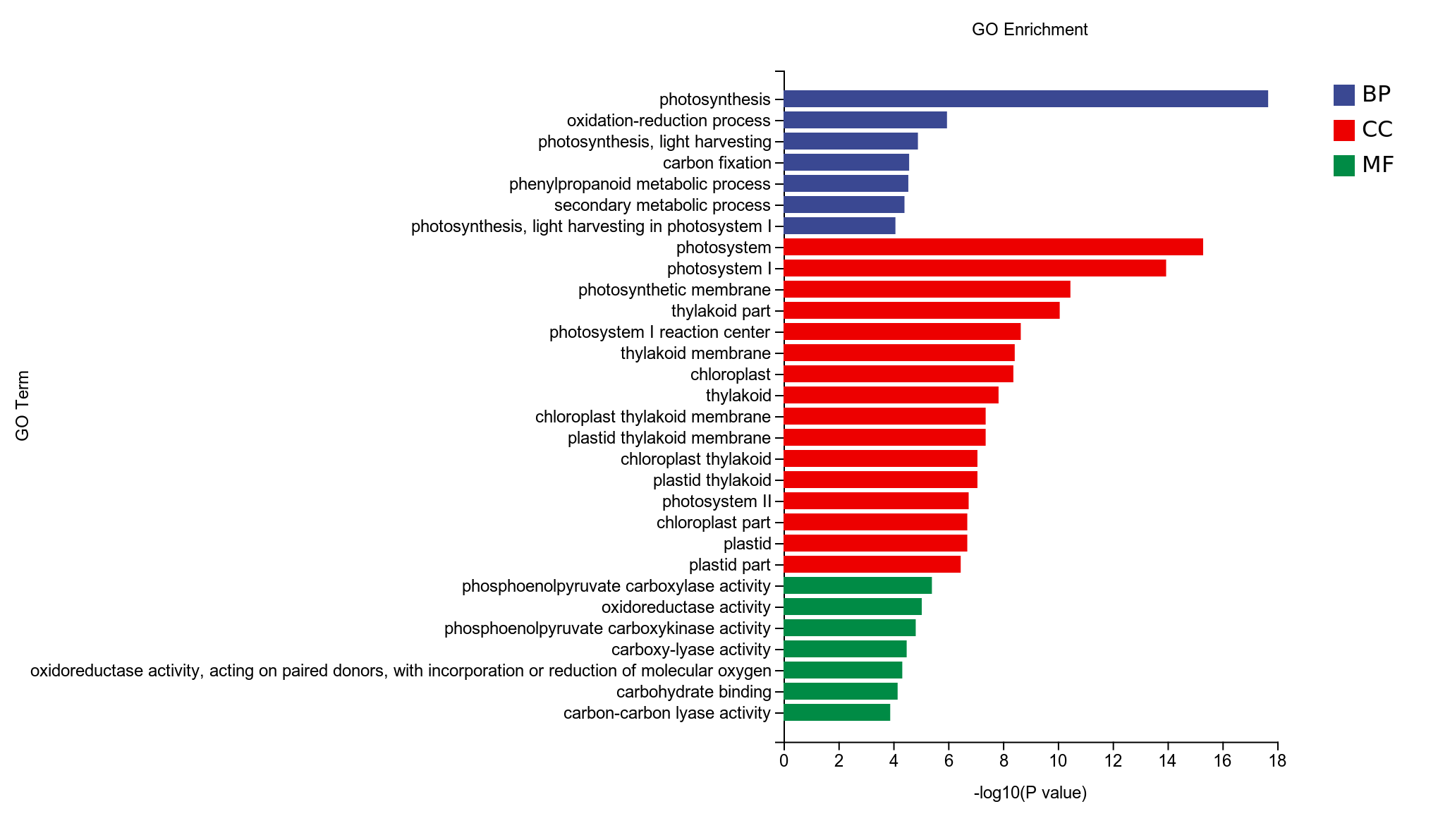


**Figure S 6**


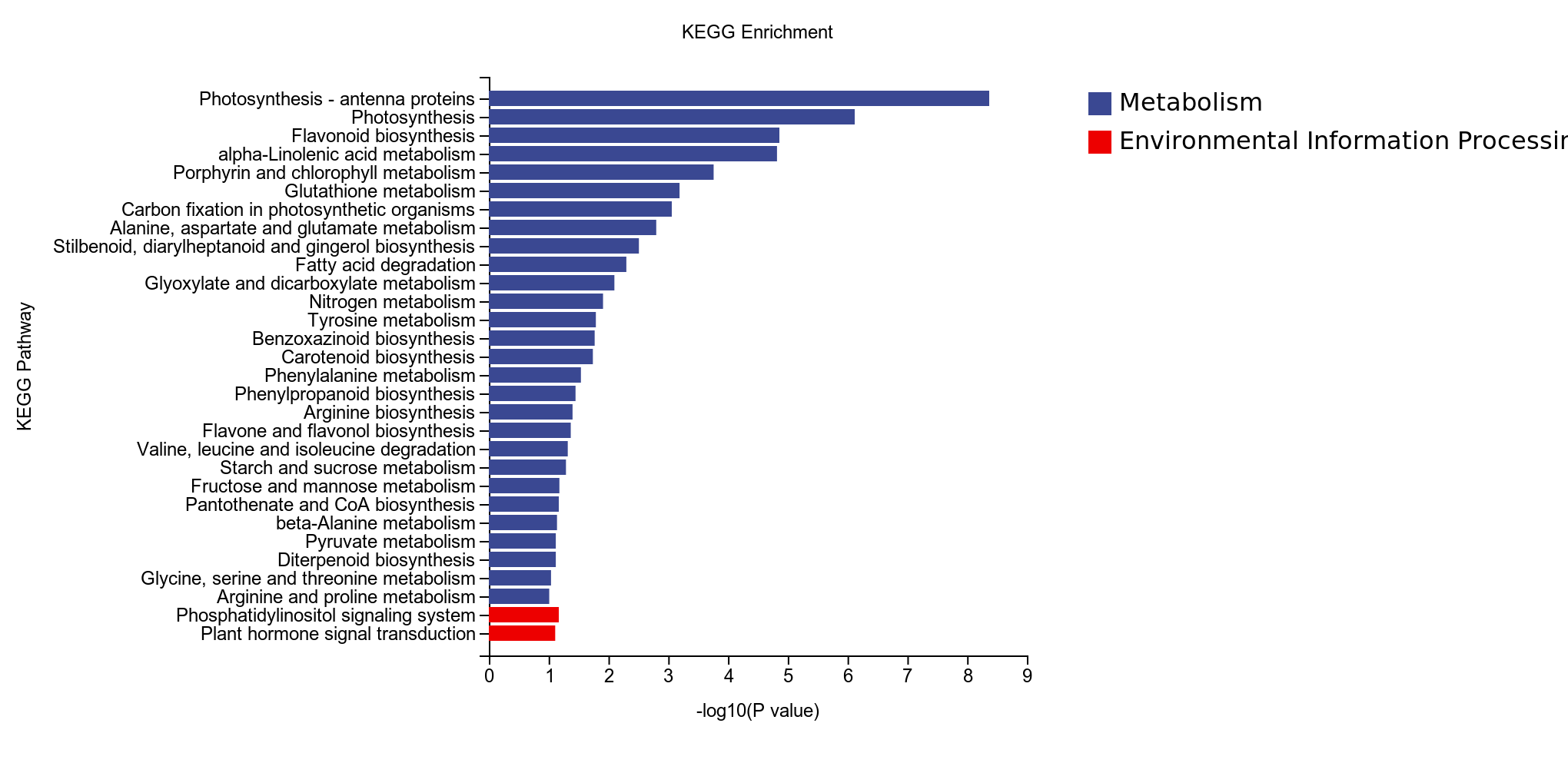


**Figure S 7**


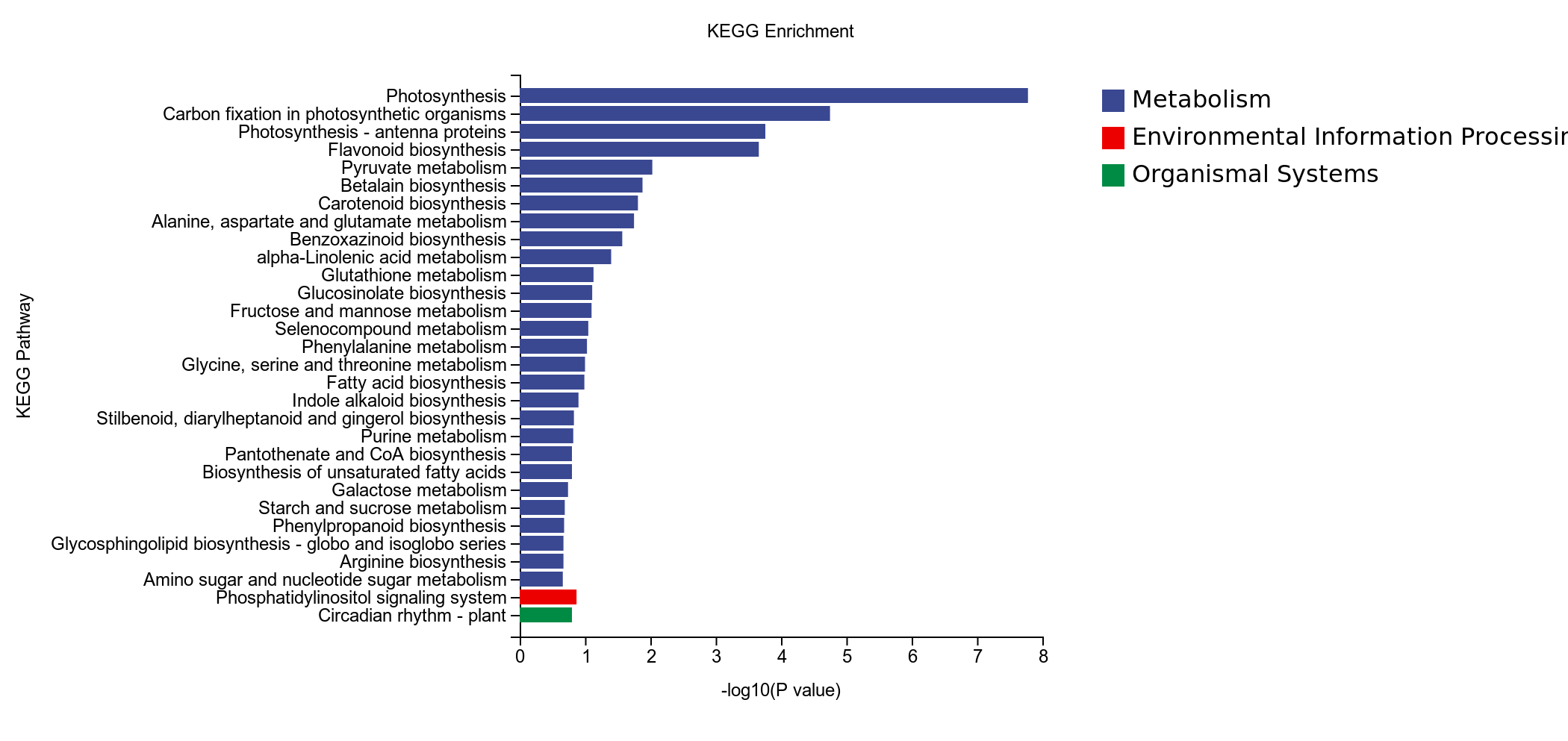

Supplement: Supplementary file 1 — Supplementary Material 1 [file 41598_2025_94385_MOESM1_ESM.docx]
